# Structural and thermodynamic basis for the recognition of the substrate-binding cleft on hen egg lysozyme by a single-domain antibody

**DOI:** 10.1038/s41598-019-50722-y

**Published:** 2019-10-29

**Authors:** Hiroki Akiba, Hiroko Tamura, Masato Kiyoshi, Saeko Yanaka, Kenji Sugase, Jose M. M. Caaveiro, Kouhei Tsumoto

**Affiliations:** 1 Department of Bioengineering, School of Engineering, The University of Tokyo, 7-3-1 Hongo, Bunkyo-ku, Tokyo 113-8656 Japan; 2 Laboratory of Pharmacokinetic Optimization, Center for Drug Design Research, National Institutes of Biomedical Innovation, Health and Nutrition, 7-6-8 Saito-Asagi, Ibaraki City, Osaka 567-0085 Japan; 3 Department of Chemistry and Biotechnology, School of Engineering, The University of Tokyo, 7-3-1 Hongo, Bunkyo-ku, Tokyo 113-8656 Japan; 4 Division of Biological Chemistry and Biologicals, National Institute of Health Sciences, 3-25-26 Tonomachi, Kawasaki-ku, Kawasaki, Kanagawa 210-9501 Japan; 5 Bioorganic Research Institute, Suntory Foundation for Life Sciences, 8-1-1, Seikadai, Seika-cho, Soraku-gun, Kyoto 619-0284 Japan; 6 Institute for Molecular Science and Exploratory Research Center on Life and Living Systems, National Institutes of Natural Sciences, 5-1 Higashiyama, Myodaiji, Okazaki, Aichi 444-8787 Japan; 7 Department of Molecular Engineering, Graduate School of Engineering, Kyoto University, Kyoto-Daigaku Katsura, Nishikyo-ku, Kyoto 615-8510 Japan; 8 Laboratory of Global Healthcare, Graduate School of Pharmaceutical Sciences, Kyushu University, 3-1-1 Maidashi, Higashi-ku, Fukuoka City, 812-8582 Japan; 9 Medical Proteomics Laboratory, The Institute of Medical Sciences, The University of Tokyo, 4-6-1 Shirokanedai, Minato-ku, Tokyo 108-8629 Japan; 10 Present Address: Astellas Pharma, Inc., 21 Miyukigaoka, Tsukuba City, Ibaraki 305-8585 Japan

**Keywords:** Proteins, X-ray crystallography, Thermodynamics, Biophysical chemistry

## Abstract

Single-domain antibodies (VHHs or nanobodies), developed from heavy chain-only antibodies of camelids, are gaining attention as next-generation therapeutic agents. Despite their small size, the high affinity and specificity displayed by VHHs for antigen molecules rival those of IgGs. How such small antibodies achieve that level of performance? Structural studies have revealed that VHHs tend to recognize concave surfaces of their antigens with high shape-complementarity. However, the energetic contribution of individual residues located at the binding interface has not been addressed in detail, obscuring the actual mechanism by which VHHs target the concave surfaces of proteins. Herein, we show that a VHH specific for hen egg lysozyme, D3-L11, not only displayed the characteristic binding of VHHs to a concave region of the surface of the antigen, but also exhibited a distribution of energetic hot-spots like those of IgGs and conventional protein-protein complexes. The highly preorganized and energetically compact interface of D3-L11 recognizes the concave epitope with high shape complementarity by the classical lock-and-key mechanism. Our results shed light on the fundamental basis by which a particular VHH accommodate to the concave surface of an antigens with high affinity in a specific manner, enriching the mechanistic landscape of VHHs.

## Introduction

Immunoglobulin molecules of the IgG family are widely employed as therapeutic agents and increasingly produced in a variety of formats as drug conjugates or bispecific antibodies^1^. In addition, fragments of antibodies and antibody-like scaffolds of small size devoid of the Fc fragment are increasingly considered as promising alternatives because of their unique pharmacokinetic profile^2^. Because of their smaller size, they are amenable to straightforward modifications such as in fusion-proteins or in drug-conjugates by protein engineering technologies. Among them, miniature single-domain antibodies derived from the variable region of the heavy chain-only antibodies of camelids have gained relevance due to their high therapeutic potential and the robust techniques employed to generate them^3,4^. Indeed, these miniature antibodies, termed VHHs, have already entered the pharmaceutical market. For example, the bivalent VHH dimer known as caplacizumab was approved in 2018 for the treatment of thrombosis in Europe^5^. VHHs are also valuable sensors for a range of applications^6,7^, in affinity chromatography^8–10^, as tools in cell-biology^11,12^, or as co-crystallization chaperons^13,14^.

The usefulness of VHHs relies on their ability to specifically recognize antigens with high affinity despite their small size. An important mechanistic feature of VHHs consists in their ability to target clefts on the surface of the antigen molecules^3,15,16^. VHHs comprise only one domain, and for that reason it is less likely that they will adopt the type of large and flat surface displayed by the variable fragments (Fvs) of conventional antibodies. Instead, because of their small size, VHHs tend to interact with the concave surface of antigen molecules^17^. The long complementarity determining region 3 (CDR3) of VHHs often protrudes from the body of the antibody and docks into clefts located on the surface of antigens with high shape complementarity, in a manner that mimics some human and bovine antibodies^15^. It is believed that in this way, VHHs compensate for the limitations of their small size, while maintaining the high affinity and specificity that constitute the hallmarks of antibodies^17^. This mechanism has been observed in a structural study that compared the binding mode of VHHs with that of Fvs using hen egg lysozyme (HEL) as a model antigen^18^. Several more studies have revealed additional details of how VHHs target the concave surface of antigen molecules^19–27^. However, the energetic contribution of each individual residue to the strength of the antibody-antigen complex has not been examined in detail, and therefore the mechanism by which VHHs favor concave surfaces of antigens is still incomplete.

Herein, we sought to reveal the molecular and energetic basis for antigen recognition by a VHH to an unprecedented level of detail. For that purpose, a VHH termed D3-L11 recognizing the active site of HEL^18^ was selected. The characteristic roles of the residues located at the antigen-antibody interface were examined using site-directed mutagenesis with high-resolution calorimetric techniques, NMR, and X-ray crystallography. We explain the fundamental basis by which D3-L11 accommodate to an antigen cleft with high affinity in a specific manner.

## Results

### Energetic basis for the antibody-antigen interaction

**Figure 1:**
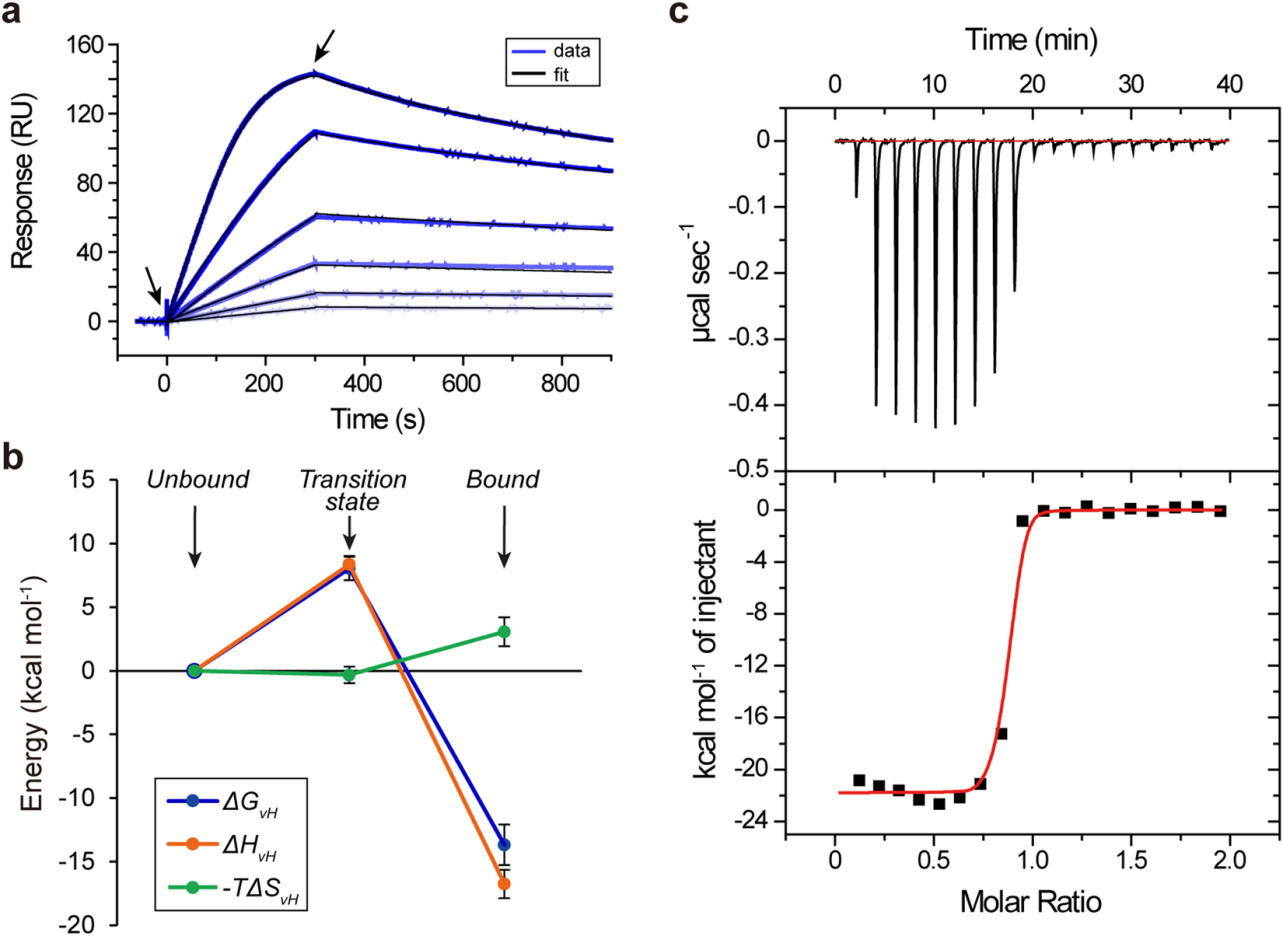
D3-L11 recognizes HEL with high affinity. (**a**) Binding of VHH to immobilized HEL at 25 °C monitored by SPR. The sensorgrams corresponded to the signal obtained from two-fold dilution series between 2 nM (darkest blue) and 0.125 nM (lightest blue). The black traces corresponded to the best fit to a 1:1 binding model. (**b**) Energy diagram of the interaction of D3-L11 with HEL with respect to the unbound state. Left, center, and right, corresponded to the unbound, transition, and equilibrium states, respectively. Errors bars corresponded to the standard error. (**c**) Titration of D3-L11 with HEL by ITC. The top panel shows the injection data (red, baseline). The bottom panel corresponded to the binding isotherm, (red, non-linear fitting). The slope of the binding isotherm was too steep for a precise estimation of the dissociation constant. In contrast, this condition favors a precise determination of the change of enthalpy.

The kinetic parameters of the interaction between the VHH antibody termed D3-L11 and its antigen HEL were determined by surface plasmon resonance (SPR) analysis at 25 °C (Fig. 1a). The values of the association rate constant (*k*_on_ = 6.1 ± 1.6 × 10^6^ M^−1^ s^−1^) and dissociation rate constant (*k*_off_ = 7.9 ± 1.7 × 10^−4^ s^−1^) were determined as described in the methods section, resulting in a dissociation constant in the sub-nanomolar range (*K*_*D*_ = 0.13 ± 0.04 nM). These values were consistent with the data reported in the literature for this antibody^18^.

Thermodynamic parameters were calculated by the van’t Hoff approximation (constant values within a narrow range of temperatures) as described in materials and methods. Binding is driven by a favorable change of enthalpy (Δ*H*_*vH*_ = −17 ± 1 kcal mol^−1^) and opposed by the change of entropy (−*T*Δ*S*_*vH*_ = 3.1 ± 1.1 kcal mol^−1^) resulting in a large and favorable change of free energy (Δ*G*_*vH*_ = −14 ± 2 kcal mol^−1^) (Supplementary Information Figs S1 and S2). The thermodynamic parameters in the transition state were determined by the Eyring approximation using the association rate constants (Supplementary Information Fig. S3). The values of change of enthalpy, change of entropy and change of free energy at the transition state were 8.4 ± 0.6 kcal mol^−1^, 0.3 ± 0.7 cal K^−1^ mol^−1^, and 8.0 ± 0.9 kcal mol^−1^, respectively. The values of all three thermodynamic parameters throughout the reaction coordinate are represented schematically in Fig. 1b.

Collectively, the interaction between D3-L11 and HEL is strongly dependent on the enthalpic component suggesting that non-covalent interactions drive the recognition of the antigen by D3-L11. The overwhelming influence of the change of enthalpy in the formation of the antibody-antigen complex was corroborated by isothermal titration calorimetry (ITC), yielding a large value for the change of enthalpy (Δ*H*° = −21.4 ± 0.6 kcal mol^−1^) (Fig. 1c). The high affinity of the complex between D3-L11 and HEL precluded a more detailed analysis of the entropy and free energy by this technique. The difference of ~4 kcal mol^−1^ between the calorimetric enthalpy and the van’t Hoff enthalpy calculated from SPR data (corresponding to a 20% of the total value) is likely related to the methodology to calculate it (a direct determination in ITC vs. the van’t Hoff method in SPR) and/or the presence of detergent in the buffer employed for SPR. Despite this modest difference, the principle that the binding is driven by the change of enthalpy and opposed by the entropy component is consistent in both techniques.

### Individual contribution of VHH’s interfacial residues to the binding affinity

To analyze the contribution of the side chains of each residue at the binding interface, first the crystal structure of the D3-L11·HEL complex was determined at 1.65 Å resolution (Supplementary Information Table S1). Overall, the crystal structure was essentially identical to that previously reported (PDB ID: 1ZVY). The root mean square deviation (RMSD) value between Cα atoms of VHH and between Cα atoms of HEL in each crystal structure as calculated with COOT^28^ was 0.49 Å and 0.54 Å, respectively (Supplementary Information Fig. S4). It is observed that the CDR3 region of D3-L11 protruded into a cleft of HEL, comprising 68% of the buried surface area of the binding interface (491 Å^2^ of a total of 719 Å^2^) with high complementarity (Sc = 0.78). The list of residues appearing at the antibody-antigen interface with a buried surface area greater than 10 Å^2^ as identified with the PISA server^29^ are listed on the Supplementary Information Table S2.

**Figure 2:**
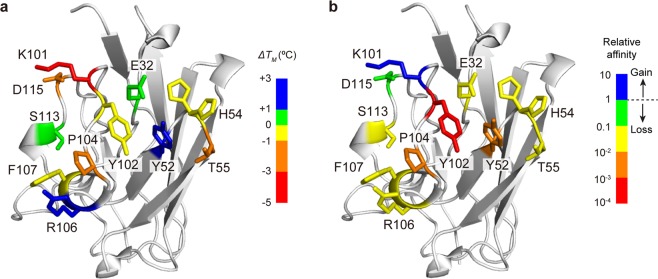
Site-directed mutagenesis. The figure shows the distribution of residues mutated to alanine. Mutated residues are depicted with sticks. (**a**) Residues are colored according to the effect of the mutation on Δ*T*_M_. Blue and red indicate greatest stabilizing and destabilizing effect, respectively. (**b**) Residues are colored depending on the effect of the mutation on the affinity, quantified with respect to WT antibody. Blue indicates a gain of affinity. Green to red signals an increasing loss of affinity.

We prepared single alanine-mutants corresponding to each of these residues (Supplementary Information Fig. S5). The structural integrity of each of the mutants was verified by circular dichroism (Supplementary Information Fig. S6). The thermal stability of D3-L11 was monitored by differential scanning calorimetry (DSC), confirming that all the mutants were stable at physiological temperatures (Supplementary Information Fig. S7). The mid-point temperature (*T*_M_) at which thermal unfolding of wild-type (WT) or mutant antibodies ocurred was determined to be in the range 62–69 °C (Table 1). The heat map in Fig. 2a identifies the locations of the residues whose Ala mutations were prepared, as a function of their effect in the thermal stability (Δ*T*_M_ = ${T}_{{\rm{M}}}^{{\rm{mutant}}}$ − ${T}_{{\rm{M}}}^{{\rm{WT}}}$). Positive and negative values indicate a gain or loss of stability, respectively. The CD and calorimetric data thus verified the integrity of the mutants for the follow-up experiments.

**Table 1:** Thermal stability of D3-L11 determined by DSC.

	*T*_*M*_ (°C)^a^	Δ*T*_*M*_ (°C) (*T*_M_^mutant^ − *T*_M_^WT^)	Order of stability (WT, reference)
WT	66.79 ± 0.01	0	0
E32A	67.26 ± 0.02	0.47	+1
Y52A	68.79 ± 0.01	2.00	+4
H54A	66.78 ± 0.01	−0.01	−1
T55A	63.74 ± 0.01	−3.05	−4
K101A	62.43 ± 0.02	−4.36	−7
Y102A	66.38 ± 0.01	−0.41	−2
P104A	63.54 ± 0.02	−3.25	−5
R106A	68.04 ± 0.01	1.25	+3
F107A	66.24 ± 0.01	−0.55	−3
S113A	67.59 ± 0.01	0.80	+2
D115A	62.92 ± 0.02	−3.87	−6

^a^Values ± S.E. of the curve fitting.

**Figure 3:**
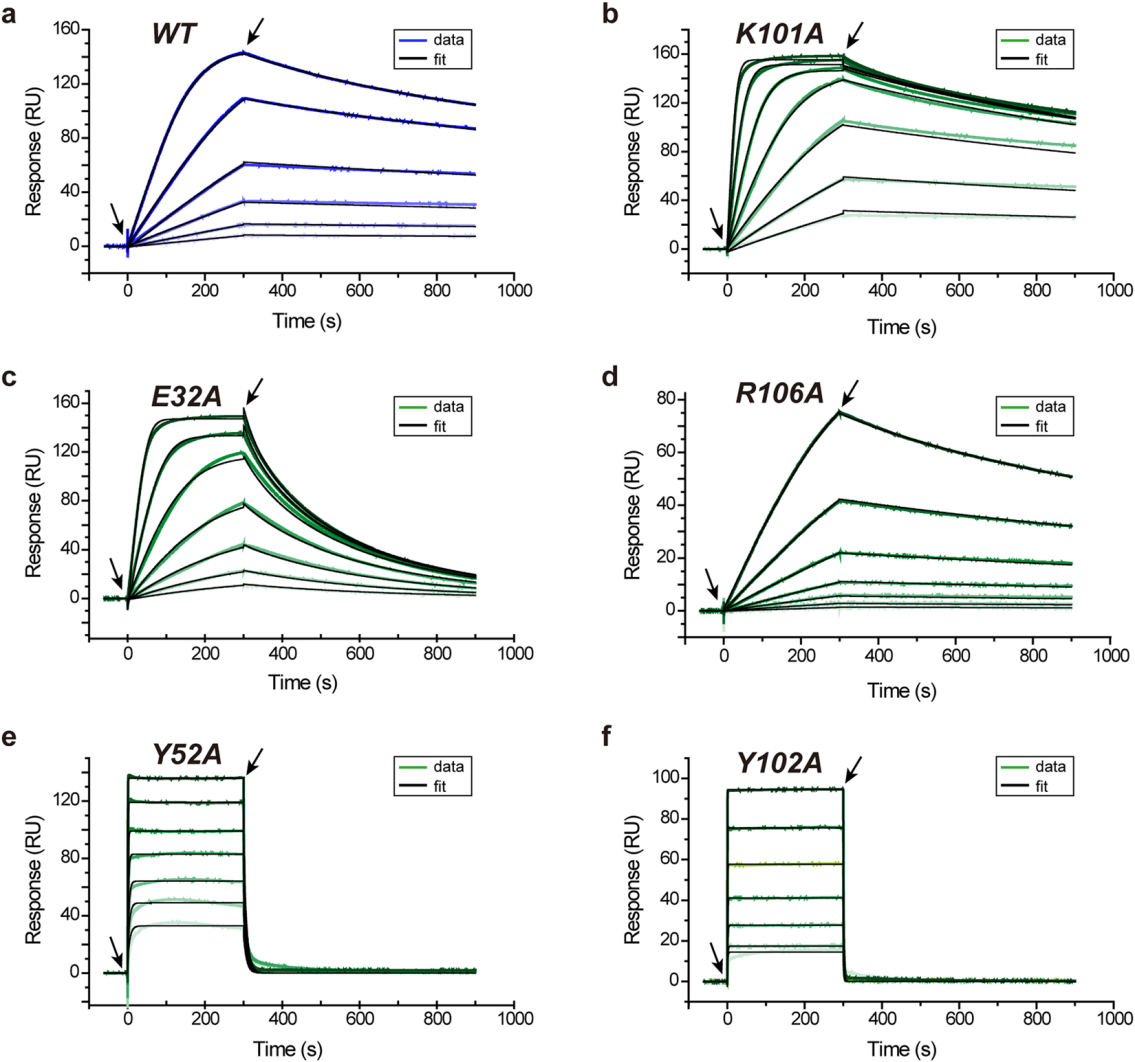
Binding of mutants. SPR sensorgrams corresponding to the binding of few D3-L11 mutants to HEL at 25 °C. Data for additional mutants are found in Supplementary Information Fig. S8. (**a**) WT (same as Fig. 1a). (**b**) K101A, corresponding to the only mutant in which the affinity increased with respect to WT. (**c**–**f**) Mutants E32A, R106A, Y52A, and Y102A showing less affinity for HEL. Experimental data is shown in blue (WT) or green (mutants). The color darkens as the concentration of VHH gradually increased in the experiment. Other details as in Fig. 1a. The concentrations employed were: (**a**) 2 nM to 0.125 nM (**b**–**d**) 16 nM to 0.25 nM, (**e**) 1024 to 16 nM, and (**f**) 2048 to 32 nM, in all cases in two-fold dilutions.

The binding interaction between each mutant and its antigen HEL was evaluated by SPR (Figs 2b and 3, Table 2, and Supplementary Information Fig. S8). Among the mutants, only K101A showed higher affinity for HEL than the WT antibody (1.4-fold tighter binding corresponding to a favorable ΔΔ*G*° = −0.2 kcal mol^−1^) despite being the mutant with the lowest stability overall (Δ*T*_M_ = −4.4 ± 0.1 °C). Except K101A, all other mutants displayed lower (or much lower) affinity for the antigen than WT antibody. From a kinetic point of view, the meager performance of these mutants was generally linked to a faster dissociation of the antibody-antigen complex (greater values of *k*_*off*_).

**Table 2:** Kinetic and thermodynamic parameters obtained from SPR.

	*k*_on_ (×10^6^ M^−1^ s^−1^)	*k*_off_ (×10^−3^ s^−1^)	*K*_D_ (nM)	Relative affinity (*K*_D_^mut^/*K*_D_^WT^)
WT^a^	6.10 ± 1.62	0.785 ± 0.170	0.129 ± 0.044	1
E32A^b^	6.43 ± 0.02	12.0 ± 0.5	1.87 ± 0.01	15
Y52A^b^	2.77 ± 0.02	128 ± 9	46.1 ± 0.5	358
H54A^b^	3.48 ± 0.01	9.21 ± 0.39	2.65 ± 0.02	21
T55A^b^	3.77 ± 0.04	11.3 ± 1.1	2.99 ± 0.04	23
K101A^b^	7.54 ± 0.01	0.687 ± 0.008	0.0911 ± 0.0002	0.7
Y102A^b^	2.32 ± 0.02	686 ± 75	295 ± 4	2,299
P104A^b^	17.4 ± 0.6	226 ± 77	13.0 ± 0.6	101
R106A^b^	1.11 ± 0.00	1.83 ± 0.05	1.65 ± 0.01	13
F107A^b^	8.99 ± 0.08	14.7 ± 1.4	1.63 ± 0.02	13
S113A^b^	5.17 ± 0.11	8.31 ± 1.6	1.61 ± 0.04	13
D115A^b^	6.11 ± 0.01	0.956 ± 0.013	0.156 ± 0.000	1.2

^a^Values ± S.D. from seven independent experiments.

^b^Values ± S.E of the fitting to the sensorgrams.

Based on the mutagenesis analysis, residues Y52, Y102 and P104 were identified as hot-spot residues bearing a large influence in binding (>100-fold loss of affinity) and therefore resulting in a large energetic contribution (ΔΔ*G* > 2.7 kcal mol^−1^). The effect of mutation in the hot-spot residues was so significant that the sensorgrams displayed a box-shape indicating a very fast dissociation rate. The values of *K*_*D*_ were also determined from their responses in equilibrium (Supplementary Information Fig. S9). The values obtained were 144 ± 22 nM, 635 ± 66 nM and 44.8 ± 19.0 nM for Y52A, Y102A and P104A, respectively. These values were uniformly greater than those obtained by global analysis by 2-3.5-fold (i.e. weaker affinity than that obtained by global analysis), but the order of affinities calculated by either method (Y102A < Y52A < P104A << others) was uniform in both calculations.

### Thermodynamic dissection of hot-spot tyrosine residues

The thermodynamic basis of the contribution of the two hot-spot tyrosine residues to the binding and to the transition state were next determined by the van’t Hoff and Eyring approximations, respectively (Supplementary Information Figs S10–S12). The thermodynamic parameters are given in Table 3. The removal of the aromatic side-chain of Tyr52 or Tyr102 resulted in large losses of change of free energy with respect to the WT antibody. In Y52A, the change of free energy was significantly diminished (Δ*G*_*vH*_ = −10 ± 1 kcal mol^−1^, corresponding to *K*_D_ = 46 ± 19 nM at 25 °C), whereas in Y102A it was even less favorable (Δ*G*_*vH*_ = −8.7 ± 4.0 kcal mol^−1^ corresponding to *K*_D_ = 413 ± 667 nM at 25 °C). The values obtained when calculations were carried out with dissociation constants obtained from the Scatchard plots were consistent with the data presented just above within experimental error (Supplementary Information Figs S13–15 and Supplementary Information Table S3).

**Table 3:** Thermodynamic binding parameters (SPR)^a^.

	Δ*G*_*vH*_	ΔΔ*G*_*vH*_^b^	Δ*H*_*vH*_	ΔΔ*H*_*vH*_^b^	−*T*Δ*S*_*vH*_	−*T*ΔΔ*S*_*vH*_^b^	Δ*C*_*p*,*vH*_	Δ*G*^‡^	Δ*H*^‡^	−*T*Δ*S*^‡^
	kcal mol^−1^	kcal mol^−1^	kcal mol^−1^	kcal mol^−1^	kcal mol^−1^	kcal mol^−1^	kcal K^−1^ mol^−1^	kcal mol^−1^	kcal mol^−1^	kcal mol^−1^
WT	−14 ± 2	—	−17 ± 1		3.1 ± 1.1	—	−0.024 ± 0.186	8.0 ± 0.9	8.4 ± 0.6	−0.32 ± 0.66
Y52A	−10 ± 1	4.0 ± 2.2	−11 ± 1	6.0 ± 1.4	0.68 ± 0.54	−2.4 ± 1.2	−0.16 ± 0.07	8.8 ± 0.2	4.3 ± 0.1	−4.5 ± 0.1
Y102A	−8.7 ± 4.0	5.3 ± 4.5	−12 ± 3	5.0 ± 3.2	3.2 ± 2.9	0.1 ± 3.1	0.15 ± 0.48	8.8 ± 1.3	6.7 ± 0.9	−2.1 ± 0.9

^a^Values ± S.E of the curve fitting based on van’t Hoff or Eyring plots.

^b^ΔΔ*G*_*vH*_ = Δ*G*_*vH*,mutant_ − Δ*G*_*vH*,WT_, ΔΔ*H*_*vH*_ = Δ*H*_*vH*, mutant_ − Δ*H*_*vH*, WT_; −*T*ΔΔ*S*_*vH*_ = (−*T*Δ*S*_*vH*, mutant_) − (−*T*Δ*S*_*vH*,WT_).

Importantly, the meager affinity between HEL and the mutated antibodies was caused by a significant loss in the change of enthalpy with respect to wild type antibody. In Y52A and Y102A the loss of enthalpy with respect to WT antibody was 6.0 ± 1.4 kcal mol^−1^, and 5.0 ± 3.2 kcal mol^−1^, respectively (Table 3). The loss of enthalpy was not compensated by a favorable change of entropy of similar magnitude, leading to weaker binding. Analogous conclusions were obtained when the analysis was carried out with the parameters from the binding at equilibrium (Supplementary Information Table S3). Furthermore, similar observations were made when the analysis was carried with the orthogonal technique of ITC (Supplementary Information Figs S16–S17 and Supplementary Information Table S4). The large loss of enthalpy with respect to WT antibody in Y52A (ΔΔ*H*° = 9.1 ± 1.4 kcal mol^−1^) and Y102A (ΔΔ*H*° = 4.8 ± 3.3 kcal mol^−1^) is consistent with the conclusion made above by SPR, although similarly to WT, the value of enthalpy obtained by ITC in mutant Y52A was slightly above the error limits of the value of enthalpy calculated by SPR.

In the transition state, the change of free energy and the change of enthalpy were unfavorable and remarkably similar to each other (Δ*G*^‡^ = 8.0 ± 0.9 kcal mol^−1^; Δ*H*^‡^ = 8.4 ± 0.6 kcal mol^−1^) (Table 3). Predictably, the change of free energy of the mutants with respect to WT is more unfavorable in the modified antibodies, suggesting that loss of affinity in equilibrium is already influenced by the events at the transition state. When the energetic terms were dissected it was observed that, contrary to the situation in equilibrium, the change of enthalpy (Δ*H*^‡^) observed in both Y52A and Y102A was stabilizing (ΔΔ*H*^‡^ = −4.1 kcal mol^−1^ and −1.7 kcal mol^−1^, respectively). In other words, the deleterious effects at this stage of the reaction coordinate in the mutants were of entropic nature, suggesting more unfavorable rearrangement of VHH and/or water molecules in the mutants than in the WT antibody.

### Structural analysis

**Figure 4:**
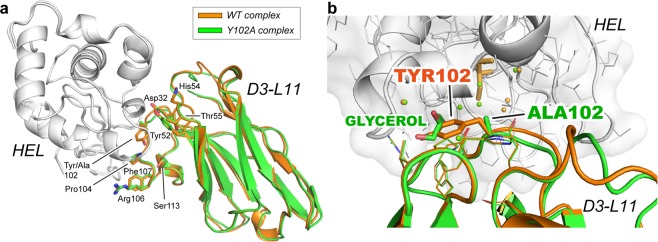
Crystal structure of the antibody-antigen complex. (**a**) Superimposed structures of the complex WT·HEL with that of the mutant Y102A·HEL. Alignment was carried out on the antigen. WT, Y102A, and HEL are shown in orange, green and gray, respectively. The side chains of residues influencing binding (loss of affinity >10-fold) are depicted with sticks. (**b**) Close-up view of the environment around Tyr102. The position of the side chains of the residues contributing to the affinity of the complex were not altered by the mutation. The vacancy generated by the removal of the side chain at position 102 was filled by a molecule of cryoprotectant (glycerol).

The large loss of affinity of Y102A (~2,300-fold) with respect to WT prompted a more detailed structural analysis by X-ray crystallography. The complex Y102A·HEL was determined at 1.50 Å and 1.55 Å resolution, respectively. The complex of the mutant with the antigen superimposed onto the complex with WT antibody is shown in Fig. 4a. A close-up view of the mutated residue and its environment is depicted in Fig. 4b. Except for the presence of a molecule of cryoprotectant (glycerol) filling up the vacancy of the side-chain of Tyr102, no other significant differences with respect to WT antibody were observed, such as in the conformation of the neighboring residues of Ala102, or in residues contributing to the affinity (Glu32, Tyr52, His54, Thr55, Pro104, Arg106 and Phe107). Similarly, the shape complementarity remained high as shown by the little change in the Sc parameter (Sc = 0.76). Although the mutation Y102A considerably impaired the binding affinity for the antigen, the conformation of the antibody-antigen interface remained largely unaffected.

**Figure 5:**
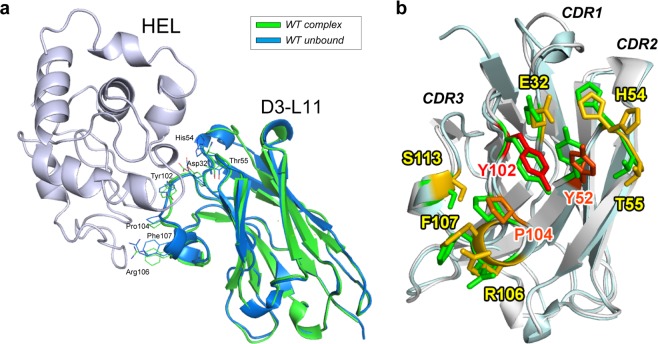
Crystal structure of unbound VHH. (**a**) Comparison of unbound D3-L11 (blue) with that in complex with HEL (green). HEL is shown in light purple. Overlaid structures using the three CDR loops. Backbone with the side chains of residues showing influence in binding (affinity loss >10-fold). (**b**) Enlarged view of the VHH binding interface from panel (a). The unbound and bound antibodies are depicted in cyan and gray, respectively; side chains of residues are shown with the same color gradient as that in Fig. 3.

The X-ray structure of WT D3-L11 in the absence of the antigen was also determined, achieving a resolution of 1.15 Å. The coordinates of unbound D3-L11 were aligned to those of the antibody in complex with HEL using the Cα atoms of the residues of the framework region (FR) (Fig. 5). The conformation of the residues contributing to the affinity did not change significantly in the unbound form compared to the antibody-antigen complex (RMSD = 0.76 Å among main chain α-carbons). Collectively, the X-ray data suggests that the interface residues of VHH contributing to binding the antigen adopted a rigid conformation.

**Figure 6:**
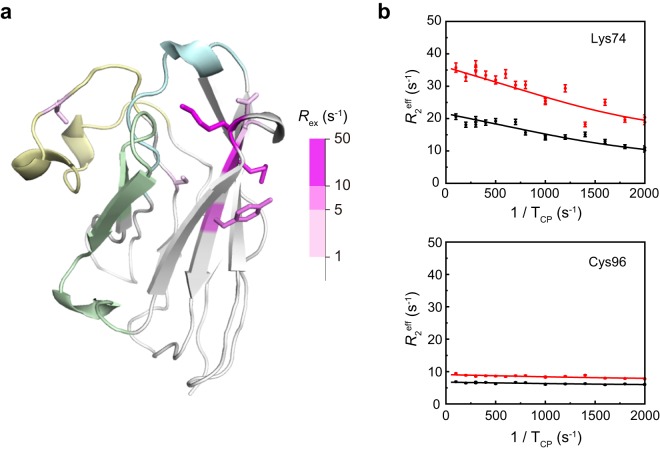
Fluctuation of residues of D3-L11 (main chain). (**a**) Structure of D3-L11 displaying the residues with the greatest fluctuations (sticks in magenta gradient). The key recognition regions CDR1, CDR2, and CDR3 are colored in cyan, green, and yellow, respectively. Other regions are shown in gray. (**b**) *R*_2_ dispersion profiles of Lys74 and Cys96 collected at 14.1 T (black) and 17.6 T (red) are shown. The residues were selected as representative examples of a residue displaying a large fluctuation, and that of a residue with no fluctuation.

To clarify the conformational fluctuation of these residues, the *R*_2_ dispersion parameters were determined by NMR. This method is employed to characterize fluctuations in the microsecond to millisecond scale, which is within the relevant time scale for protein function. Only six residues displayed values of *R*_ex_ greater than 1 s^−1^ (Lys74, Asn77, Met78, Tyr80, Cys96 and Val103) (Fig. 6). Those residues with significant fluctuations (*R*_ex_ > 5 s^−1^) were exclusively located in the FR3 (Lys74, Met78, Tyr80). Except for Val103, residues in the CDRs showed little fluctuation, suggesting that the CDRs were little dynamic in solution, in agreement with the conclusions extracted from the crystallographic data.

## Discussion

The combination of thermodynamic, structural and dynamic information is a powerful tool to accurately describe interaction phenomena like that in the recognition of antigens by antibodies. Herein we report that the recognition mode of D3-L11 for its antigen HEL shared some features in common with Fv from conventional antibodies^30^ and some features in common with other VHHs^31,32^. The present study further dissected the roles of each residue at the binding interface by alanine-scan mutagenesis.

Previous studies have suggested a dominant role for the CDR3 in the recognition of antigens by antibodies of the VHH class^17^. The CDR3 region stems from recombination of all of V, D and J gene segments, and somatic hypermutation, becoming the most diverse CDR in length and sequence in antibodies^33^. The analysis of D3-L11 carried herein supports this general idea, and embraces the concept of three-dimensional proximity around a core of key residues acting as energetic hot-spots. Of the three hot-spots residues identified in D3-L11, two of them are located in the CDR3 and one in the CDR2. These three residues are clustered together in the three-dimensional structure, and uniformly surrounded by a constellation of residues of lesser but still appreciable importance for binding the antigen. This uniform layer comprises residues belonging to each of the three CDR regions of the VHH.

This kind of arrangement is commonly observed in the paratope of Fvs and in the interaction surface of protein-protein complexes^30,34–36^. However, an important difference between Fvs and D3-L11 is observed. Whereas Fvs generally recognize planar epitopes^37–39^, D3-L11 recognize a cleft on the surface of the antigen with high shape complementarity (Sc > 0.75), a signature characteristic of numerous other VHHs^18^. The results presented herein demonstrated that hot-spot residues coalescing on a small geometric region are characteristic not only when interacting with the planar surface of antigens such as in Fvs, but can also occur in a VHH recognizing a concave surface of the antigen.

In contrast, this arrangement of residues is not common among other VHHs. Generally, VHHs of known structure display long, extended CDR3 (or less commonly, CDR1)^40^ that interact with the antigen epitope independently from other regions of the VHH moiety^21,23–25^. For example, a previous study of a VHH exhibiting a protruding CDR3 indicated that the most determining residues were present in the extended CDR3, but not outside of it^21^. The constellation of residues of VHH in that study is clearly different from that of D3-L11, even if there are also some similarities in other parts of the CDR3. In the majority of VHHs with long CDR3, and D3-L11 is not an exception, this loop covers a relatively hydrophobic surface of the framework region 2 (FR2) of the VHH molecule. These miniature antibodies utilize the long CDR3 loop to naturally act as a part of the single domain antibody, rather than as an independent element. The equivalent surface in Fv is located between the VL and VH regions^41^.

The structural data showed only minor differences in the conformation of the main chain between unbound and bound D3-L11, suggesting that preorganization of the interaction surface is a key aspect of the mechanism of recognition. The rigidity of the residues comprising the interaction surface would ensure a highly cooperative mode of interaction of the three CDR loops, enhancing the affinity for the antigen. Similar structural characteristics were observed for a VHH interacting with a shallow concave surface of its antigen^26^. In contrast, in VHHs where CDR3 protrudes to access deep concave sites the structural data revealed large differences between the complexed and unbound structures^21,27^. Mixed of VHHs binding concave sites of antigens without protruding CDRs show slight reorganization of the residues of these CDRs loops upon binding^20,22,27^. In our case, because both HEL^42^ and D3-L11 (this study) are rigid, the interaction could be explained with classical mechanism of lock-and-key^43^. The results of thermodynamic analysis including dominant enthalpic component throughout the association process and reduced enthalpy upon hot-spot mutagenesis are consistent with this model. Despite the little structural change of the interface residues, binding is strongly opposed by the change of entropy suggesting an intricate role for the solvent.

In conclusion, the paratope of the VHH antibody D3-L11 recognized its epitope by a lock-and-key mechanism, and is organized similarly to that of a conventional antibody (Fvs). Although this mechanism is not common among VHHs, the high affinity of D3-L11 indicates that mimicking Fvs may also work for a VHH to achieve its high affinity and specificity, the two hallmarks of antibodies. Because the long CDR3 covers the surface that emerges in the absence of the VL domain of IgGs, diversity in this loop is fully employed to generate a preorganized interface on the convex surface of the VHH. This organization is suitable to recognize concave surface of antigens, complementing the more common mechanism of protrusion of CDR observed in other VHHs. Diversity in binding modes enable VHHs to accommodate to various antigen molecules and thus brings VHHs advantages over other cleft binders, achieving high affinity and specificity.

## Materials and Methods

### Cloning, expression and purification of D3-L11

A synthetic gene of D3-L11 optimized for *Escherichia coli* (Genscript) was cloned in pRA2^44^ between NcoI and SacII restriction sites. The construct also contained a *pelB* signal peptide at the N-terminus, and a His_6_-tag at the C-terminus. For expression, *E*. *coli* strain BL21(DE3) carrying the expression vector of D3-L11 were grown in 1 L of LB medium containing 50 μg/mL ampicillin at 28 °C and 120 rpm. Expression was induced by addition of 0.5 mM isopropyl-β-D-thiogalactopyranoside when the optical density at 600 nm reached 0.5 after which the temperature was reduced to 20 °C overnight. The cells were harvested by centrifugation (7,000 × *g* for 15 min) at 4 °C. The cell pellet was resuspended in buffer A (20 mM TRIS-HCl, 500 mM NaCl, pH 8.0) supplemented with 5 mM imidazole, after which it was lysed with an ultrasonic disruptor (UD-201, TOMY) for 15 min. The cell lysate was centrifuged (40,000 × *g* for 30 min) at 4 °C. The supernatant was filtered through a membrane of a nominal pore size of 0.45 μm and subsequently loaded onto a 1 mL of Ni-NTA agarose column (Qiagen) equilibrated with buffer A. After a washing step with Buffer A containing 100 mM imidazole, VHHs were eluted from the column with buffer A supplemented with 500 mM imidazole. The eluate was dialyzed against buffer A, and subjected to size-exclusion chromatography (SEC) using a HiLoad 26/600 superdex 75 pg column (GE Healthcare) equilibrated with a buffer containing 20 mM TRIS-HCl, 150 mM NaCl, and 1 mM EDTA at pH 7.4.

For crystallization of the unbound form of D3-L11, the gene encoding the antibody was cloned into a Champion pET-SUMO vector bearing a His_6_-SUMO-tag. The protein was expressed as above. After the affinity chromatography step, the His_6_-SUMO-tag was cleaved-off with Ulp1 protease overnight at 4 °C in 20 mM TRIS-HCl, 150 mM NaCl at pH 8.0. The protein was separated from the protease, from the cleaved tag, and from the uncleaved protein by immobilized metal-affinity chromatography. The flow-thorough was concentrated and subjected to SEC using a HiLoad 16/600 superdex 75 pg column as described above.

### Preparation of the antigen

HEL was purchased from Wako Pure Chemical (Cat. No. 126-02671, Japan) and solubilized in phosphate-buffered saline (PBS; 137 mM NaCl, 2.7 mM KCl, 10 mM Na_2_HPO_4_, 1.8 mM KH_2_PO_4_, pH 7.4) at the desired concentration and used without further purification.

### Circular dichroism

The secondary structure of D3-L11 was examined in a CD J-820 spectrometer (Jasco, Japan) with a 1-mm quartz cuvette. Measurements were performed in a buffer containing 20 mM TRIS-HCl, 150 mM NaCl, 1 mM EDTA, pH 7.4 at a protein concentration of 10 μM. The spectrum of each sample was recorded five times at a speed of 50 nm/min and at 25 °C.

### Differential scanning calorimetry

Thermal stability of D3-L11 and mutants (20 μM) was monitored with a VP-Capillary DSC instrument (MicroCal) in PBS. Samples were scanned at a speed of 1 °C/min from 10 to 100 °C. Data analysis was performed with Origin 7.0 using a non-two-state denaturation model.

### Surface plasmon resonance

The binding of D3-L11 to HEL was determined by SPR using a Biacore T200 instrument (GE Healthcare). HEL (6.5 μg/mL) in 10 mM sodium acetate at pH 5.5 was immobilized onto a Series S CM5 Sensor Chip at 200 RU as previously described^45,46^. Measurements were performed at 25 °C in PBS buffer supplemented with the detergent Tween 20 (0.005%). Contact and dissociation times were 300 and 600 sec, respectively. The range of concentrations of D3-L11 was adjusted depending on the mutation studied and indicated where appropriate. Regeneration was conducted with 1.0 M Arg-HCl at pH 4.4 for 60 sec. To obtain the thermodynamic parameters the experiments were conducted at several temperatures. Data analysis was conducted with the instrument’s BiaEvaluation Software. Global fitting analysis and Scatchard plots were performed with a 1:1 binding model. The thermodynamic parameters were determined with the BiaEvaluation Software employing non-linear regressions (van’t Hoff plot) or linear regressions (Eyring plots). The van’t Hoff analysis of WT was not conducted with the data obtained from the Scatchard plot because the association step did not reach a plateau under the experimental conditions tested. In the experiments with the mutants at 30 °C, non-specific binding significantly affected the sensorgrams, and thus these data were excluded from the analysis.

### Isothermal titration calorimetry

ITC measurements were carried out in an iTC200 instrument (Microcal) at 25 °C and a stirring rate of 1000 rpm in PBS buffer. The WT antibody or its mutants (10 μM) were titrated with HEL (100 μM) by injecting one-time 0.5 μL followed by 18 injections of 2.0 μL each with a fixed time between injections of 120 sec. The binding isotherm was fitted by non-linear regression with the program Origin 7.0 to a 1:1 binding model to obtain the thermodynamic parameters of the interaction.

### Crystallization

For the crystallization of the antibody·antigen complexes, WT or Y102A antibody was mixed with ca. 5-fold excess of HEL in PBS, followed by dialysis against a buffer containing 20 mM TRIS-HCl, 100 mM NaCl, pH 8.0 overnight. The antibody-antigen complex was separated from excess HEL by SEC using a HiLoad 16/600 superdex 75 pg column equilibrated in the same buffer. The protein sample was concentrated to 13 mg/mL (WT-HEL complex) or 8.3 mg/mL (Y102A-HEL complex) in an Amicon Ultra-4 unit (molecular weight cutoff 10 kDa). Preliminary crystallization experiments were explored by mixing an equal volume of precipitant solution and protein solution in an Oryx8 instrument (Douglas Instruments) using commercial sparse matrix screens (Hampton Research) at 20 °C. Each complex (WT·HEL or Y102A·HEL) was crystallized in a solution containing 100 mM sodium nitrate and 16% PEG-3350 (pH 8.0). The complex between HEL and the mutant Y102A was independently crystalized in a buffer containing 100 mM lithium chloride and 18% PEG-3350. Crystals of WT D3-L11 (10 mg/mL) in the unbound form were obtained in a solution containing 100 mM Tris-HCl and 2.15 M ammonium sulfate at pH 7.0.

### Data collection and refinement

Data were collected in beamlines BL5A and AR-NW12 at the Photon Factory (Tsukuba, Japan) under cryogenic conditions (100 K). The diffraction images were processed with the program MOSFLM and subsequently merged and scaled with the program SCALA of the CCP4 suite^47^. The structures were determined using the method of molecular replacement with the program PHASER^48^. The coordinates were refined with the program REFMAC5^49^, and manually improved with COOT^28^. Validation was carried out with PROCHECK^50^. No residues were found in the disallowed regions of the Ramachandran plot. Residues were numbered sequentially. Kabat sequence numbering is shown in Supplementary Information Table S5. Data collection and refinement statistics are given in Supplementary Information Table S1.

### *R*_2_ dispersion (NMR)

Three-dimensional spectra of HNCO, HN(CA)CO, HNCA, HN(CO)CA, HNCACB, and HN(CO)CACB were measured on an AVANCE DRX600 spectrometer (Bruker BioSpin) for sequential assignments of the backbone ^1^H, ^13^C, and ^15^N chemical shifts^51^ of free D3-L11 using the protein dissolved at 0.5 mM in NMR buffer (95% H_2_O/5% D_2_O, 20 mM sodium phosphate [pH 7.4], 143 mM NaCl). NMR data were processed and analyzed as previously described^52^. ^15^N effective *R*_2_ relaxation rates were measured at 37 °C on AVANCE DRX600 and AVANCE DMX750 spectrometers (Bruker BioSpin) using the ^1^H continuous-wave Carr-Purcell-Meiboom-Gill (CPMG) pulse sequence^53^. Effective *R*_2_ rates were calculated as described previously^54^. Relaxation dispersion data whose *R*_2_ values changed by <1 s^−1^ over the entire range of τ_cp_ were excluded. By using the program GLOVE^55^, the relaxation dispersion curves were fitted to a two-state exchange model.

### Accession numbers

The coordinates and structure factors of unbound D3-L11 (entry code 6JB9), WT D3-L11 in complex with HEL (entry code 6JB8), and Y102A D3-L11 in complex with HEL (entry codes 6JB2 and 6JB5) have been deposited in the Protein Data Bank.

## Supplementary information

Supplementary Information
