# Supplementary material for: Structural and thermodynamic basis for the recognition of the substrate-binding cleft on hen egg lysozyme by a single-domain antibody
Source: Sci Rep. 2019 Oct 29;9:15481. doi: 10.1038/s41598-019-50722-y (PMC6820745; doi:10.1038/s41598-019-50722-y)
Supplement: Supplementary file 1 — Supplementary Information [file 41598_2019_50722_MOESM1_ESM.pdf]

Supplementary Information: Structural and thermodynamic basis for the recognition of the substrate-binding cleft on hen egg lysozyme by a single-domain antibody.

Hiroki Akiba,[1,2†] Hiroko Tamura,[3†‡] Masato Kiyoshi,[4] Saeko Yanaka,[5,6] Kenji Sugase,[5,7] Jose M. M. Caaveiro,[1,8,*] and Kouhei Tsumoto[1,2,3,9,*]

[1]Department of Bioengineering, School of Engineering, The University of Tokyo, 7-3-1 Hongo, Bunkyo-ku, Tokyo, 113-8656 Japan. [2]Laboratory of Pharmacokinetic Optimization, Center for Drug Design Research, National Institutes of Biomedical Innovation, Health and Nutrition, 7-6-8 Saito-Asagi, Ibaraki City, Osaka, 567-0085 Japan, [3]Department of Chemistry and Biotechnology, School of Engineering, The University of Tokyo, 7-3-1 Hongo, Bunkyo-ku, Tokyo, 113-8656 Japan. [4]Division of Biological Chemistry and Biologicals, National Institute of Health Sciences, 3-25-26 Tonomachi, Kawasaki-ku, Kawasaki, Kanagawa, 210-9501 Japan. [5]Bioorganic Research Institute, Suntory Foundation for Life Sciences, 8-1-1, Seikadai, Seika-cho, Soraku-gun, Kyoto, 619-0284 Japan. [6]Institute for Molecular Science and Exploratory Research Center on Life and Living Systems, National Institutes of Natural Sciences, 5-1 Higashiyama, Myodaiji, Okazaki, Aichi, 444-8787 Japan. [7]Department of Molecular Engineering, Graduate School of Engineering, Kyoto University, Kyoto-Daigaku Katsura, Nishikyo-ku, Kyoto 615-8510, Japan. [8]Laboratory of Global Healthcare, Graduate School of Pharmaceutical Sciences, Kyushu University, 3-1-1 Maidashi, Higashi-ku, Fukuoka City, 812-8582 Japan. [9]Medical Proteomics Laboratory, The Institute of Medical Sciences, The University of Tokyo, 4-6-1 Shirokanedai, Minato-ku, Tokyo, 108-8629 Japan.

[†] These authors contributed equally to the work.

[‡] Present address: Astellas Pharma, Inc., 21 Miyukigaoka, Tsukuba City, Ibaraki, 305-8585 Japan

*Correspondence should be addressed to Jose M. M. Caaveiro (jose@phar.kyushu-u.ac.jp) or Kouhei Tsumoto (tsumoto@bioeng.t.u-tokyo.ac.jp).

Supplementary Information Table S1: Data collection and refinement statistics.

Statistical values given in parenthesis refer to the highest resolution bin.

**Data Collection**	**WT + HEL**	**Y102A + HEL[1]**	**Y102A + HEL[2]**	**WT (unbound)**
Space Group	P 1 2_1 1	P 1 2_1 1	P 1 2_1 1	P 1 2_1 1
Unit cell				
a, b, c (Å)	40.2, 49.5, 59.3	44.0, 49.4, 58.0	44.0, 49.4, 57.9	26.2, 53.4, 36.2
α, β, γ (°)	90.0, 93.4, 90. 0	90.0, 93.7, 90.0	90.0, 93.8, 90.0	90.0, 91.5, 90.0
Resolution (Å)	40.2 - 1.65	34.0 - 1.50	49.4 - 1.55	26.7 - 1.15
Wavelength	1.0000	1.0000	1.0000	1.0000
Observations	91,977 (7,725)	205,482 (19,140)	182,442 (22,258)	181,269 (19,293)
Unique reflections	26,392 (2,783)	39,075 (4,911)	26,086 (5,168)	34,665 (4,414)
$R_{merge.}$	0.076 (0.399)	0.085 (0.480)	0.094 (0.594)	0.070 (0.210)
$R_{p.i.m.}$	0.047 (0.294)	0.040 (0.269)	0.045 (0.313)	0.033 (0.110)
$CC_{1/2}$	0.997 (0.848)	0.996 (0.804)	0.996 (0.717)	0.997 (0.952)
$I / \sigma (I)$	11.1 (2.2)	11.9 (2.8)	10.8 (2.4)	14.1 (6.0)
Multiplicity	3.5 (2.8)	5.3 (3.9)	5.1 (4.3)	5.2 (4.4)
Completeness (%)	93.7 (68.0)	97.8 (85.0)	99.7 (98.0)	97.9 (85.9)
**Refinement**				
Resolution (Å)	40.2 - 1.65	34.0 - 1.50	49.4 - 1.55	26.7 - 1.15
R_{work} / R_{free} (%)	16.7 / 20.7	12.5 / 16.5	13.2 / 16.9	10.4 / 13.2
No. protein chains	1 : 1	1 : 1	1 : 1	1
No. atoms				
Protein	1,994	2,021	2,020	1,044
Other	20	16	16	20
Water	279	307	310	211
B-factor ($Å^2$)				
Protein	28.8	16.2	16.3	7.2
Others	34.8	23.3	22.4	19.2
Water	35.1	28.2	28.3	18.5
Ramachandran				
Preferred (%)	91	89.2	91.4	94.6
Allowed (%)	9.1	10.8	8.6	5.4
Outliers (%)	0	0	0	0
RMSD bond (Å)	0.019	0.015	0.014	0.014
RMSD angle (°)	1.81	1.57	1.58	1.76
PDB Id	6JB8	6JB2	6JB5	6JB9

[1] Crystals obtained in mother liquor containing 100 mM $NaNO_3$ and 16% PEG 3,350.

[2] Crystals obtained in mother liquor containing 100 mM LiCl and 18% PEG 3,350.

Supplementary Information Table S2. Buried residues at the antibody-HEL interface (BSA > 10 $Å^2$).

Position			Contact interface		
**CDR**	**Sequential**	**Kabat**	**BSA ($Å^2$)**	**Fraction buried**[a]	**Polar contacts**[b]
**CDR1**	E32	E32	33.4	1.0	H, S
**CDR2**	Y52	Y52	32.7	1.0	H
	H54	H53	55.5	0.60	
	T55	T54	31.4	0.35	H
	K101	K97	91.8	0.57	H, S
	Y102	Y98	92.9	0.76	H
	P104	P100	65.2	0.98	
**CDR3**	R106	R100b	38.7	0.19	
	F107	F100c	77.7	0.80	
	S113	S100i	20.6	0.92	
	D115	D101	32.0	0.34	H, S

[a]Fraction of BSA with respect to ASA.

[b]H, hydrogen bond; S, salt bridge.

Supplementary Information Table S3. Thermodynamic parameters of mutants obtained by SPR (Scatchard analysis).

	ΔG_{vH}	$\Delta\Delta G_{vH}$[a]	ΔH_{vH}	$\Delta\Delta H_{vH}$[a]	$-T\Delta S_{vH}$	$\Delta(-T\Delta S_{vH})$[a]	$\Delta C_{p,vH}$
	kcal mol^{-1}	kcal mol^{-1}	kcal mol^{-1}	kcal mol^{-1}	kcal mol^{-1}	kcal mol^{-1}	kcal K^{-1}mol^{-1}
**Y52A**	−9.5 ± 0.1	(4.5 ± 2.0)	−7.9 ± 0.1	(9.1 ± 1.0)	−1.6 ± 0.1	(−4.7 ± 1.1)	−0.11 ± 0.01
**Y102A**	−8.5 ± 0.4	(5.5 ± 2.0)	−10.7 ± 0.3	(6.3 ± 1.0)	2.3 ± 0.3	(−0.8 ± 1.1)	0.33 ± 0.05

[a]The reference value of WT protein is given in Table 3 of the manuscript.

Supplementary Information Table S4. Thermodynamic parameters from ITC measurements.[a]

	n	$\Delta G°$	$\Delta\Delta G°$ [b]	K_D	$\Delta H°$	$\Delta\Delta H°$ [b]	$-T\Delta S°$	$\Delta(-T\Delta S°)$ [b]
		kcal mol^{-1}	kcal mol^{-1}	μM	kcal mol^{-1}	kcal mol^{-1}	kcal mol^{-1}	kcal mol^{-1}
**WT**	0.88 ± 0.07	−11.5 ± 0.9		0.0038 ± 0.0055	−21.4 ± 0.6		9.9 ± 1.0	
**Y52A**	0.93 ± 0.13	−9.2 ± 0.5	2.3 ± 1.0	1.0 ± 0.3	−12.3 ± 1.3	9.1 ± 1.4	3.1 ± 1.4	−6.8 ± 1.7
**Y102A**	1.23 ± 0.28	−8.2 ± 0.2	3.3 ± 0.9	1.8 ± 1.5	−16.6 ± 3.2	4.8 ± 3.3	8.4 ± 3.2	−1.5 ± 3.3

[a] Values ± SD from two independent measurements. [b] $\Delta\Delta Q° = \Delta Q°_{mutant} - \Delta Q°_{WT}$ (Q: any thermodynamic parameter).

Supplementary Information Table S5. Definition of CDR and framework regions (FR).

Region	Residue	Sequential	Kabat
FR1	D	1	1
	V	2	2
	Q	3	3
	L	4	4
	V	5	5
	E	6	6
	S	7	7
	G	8	8
	G	9	9
	G	10	10
	S	11	11
	V	12	12
	Q	13	13
	A	14	14
	G	15	15
	G	16	16
	S	17	17
	L	18	18
	R	19	19
	L	20	20
	S	21	21
	C	22	22
	A	23	23
	A	24	24
	S	25	25
**CDR1**	**G**	26	26
	**S**	27	27
	**T**	28	28
	**D**	29	29
	**S**	30	30
	**I**	31	31
	**E**	32	32
	**Y**	33	33
	**M**	34	34
	**T**	35	35
FR2	W	36	36
	F	37	37
	R	38	38
	Q	39	39
	A	40	40
	P	41	41
	G	42	42
	K	43	43
	A	44	44
	R	45	45
FR2	E	46	46
	G	47	47
	V	48	48
	A	49	49
**CDR2**	**A**	50	50
	**L**	51	51
	**Y**	52	52
	**T**	53	52a
	**H**	54	53
	**T**	55	54
	**G**	56	55
	**N**	57	56
	**T**	58	57
	**Y**	59	58
	**Y**	60	59
	**T**	61	60
	**D**	62	61
	**S**	63	62
	**V**	64	63
	**K**	65	64
	**G**	66	65
FR3	R	67	66
	F	68	67
	T	69	68
	I	70	69
	S	71	70
	Q	72	71
	D	73	72
	K	74	73
	A	75	74
	K	76	75
	N	77	76
	M	78	77
	A	79	78
	Y	80	79
	L	81	80
	R	82	81
	M	83	82
	D	84	82a
	S	85	82b
	V	86	82c
	K	87	83
	S	88	84
	E	89	85
	D	90	86
FR3	T	91	87
	A	92	88
	I	93	89
	Y	94	90
	T	95	91
	C	96	92
	G	97	93
	A	98	94
**CDR3**	**T**	99	95
	**R**	100	96
	**K**	101	97
	**Y**	102	98
	**V**	103	99
	**P**	104	100
	**V**	105	100a
	**R**	106	100b
	**F**	107	100c
	**A**	108	100d
	**L**	109	100e
	**D**	110	100f
	**Q**	111	100g
	**S**	112	100h
	**S**	113	100i
	**Y**	114	100j
	**D**	115	101
	**Y**	116	102
FR4	W	117	103
	G	118	104
	Q	119	105
	G	120	106
	T	121	107
	Q	122	108
	V	123	109
	T	124	110
	V	125	111
	S	126	112
	S	127	113

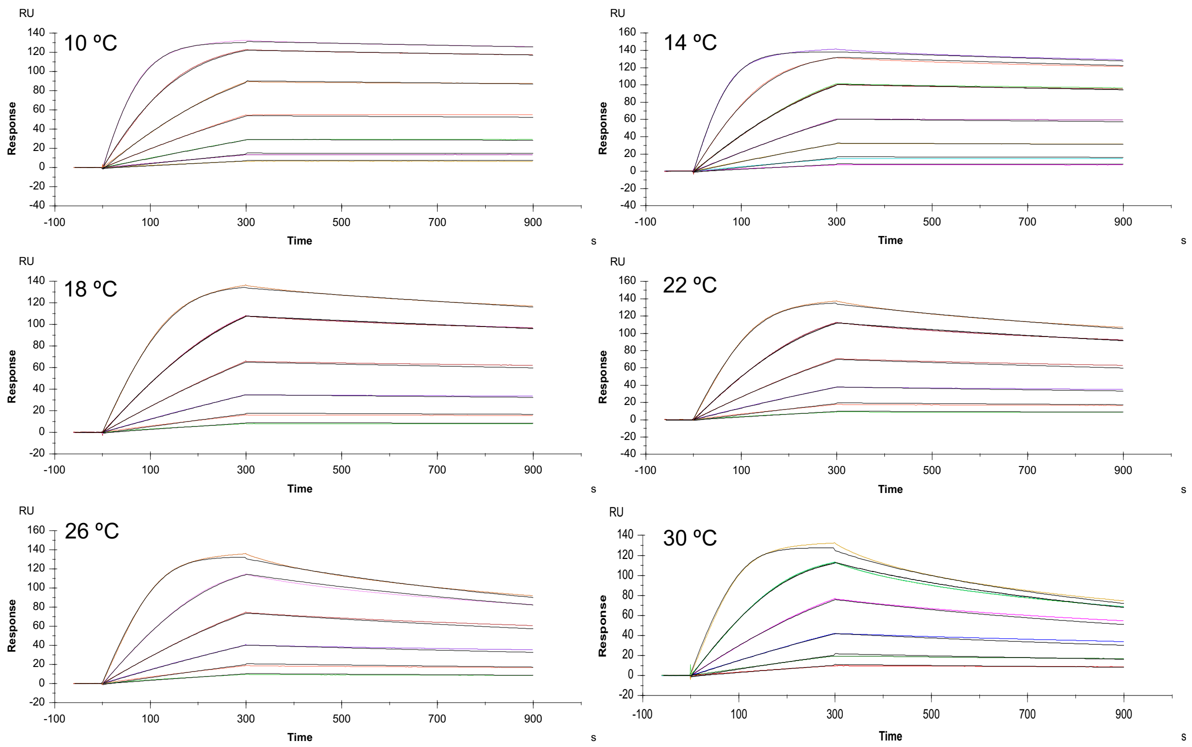

Temperature (ºC)	k_{on} ($\times 10^6$ M^{-1} s^{-1})	k_{off} ($\times 10^{-4}$ s^{-1})	K_D (nM)
10	2.81 ± 0.00	0.752 ± 0.004	0.0267 ± 0.0001
14	3.52 ± 0.00	1.39 ± 0.00	0.0395 ± 0.0000
18	4.99 ± 0.00	2.74 ± 0.00	0.0549 ± 0.0001
22	5.48 ± 0.00	4.92 ± 0.01	0.0897 ± 0.0001
26	6.81 ± 0.01	8.47 ± 0.01	0.127 ± 0.000
30	8.48 ± 0.02	15.0 ± 0.0	0.177 ± 0.000

Supplementary Information Figure S1. Interaction between D3-L11 and HEL monitored at various temperatures using SPR (multi-cycle method). The values of the association and dissociation rates, and those of the dissociation constants are given in the lower panel with standard errors of the curve fitting.

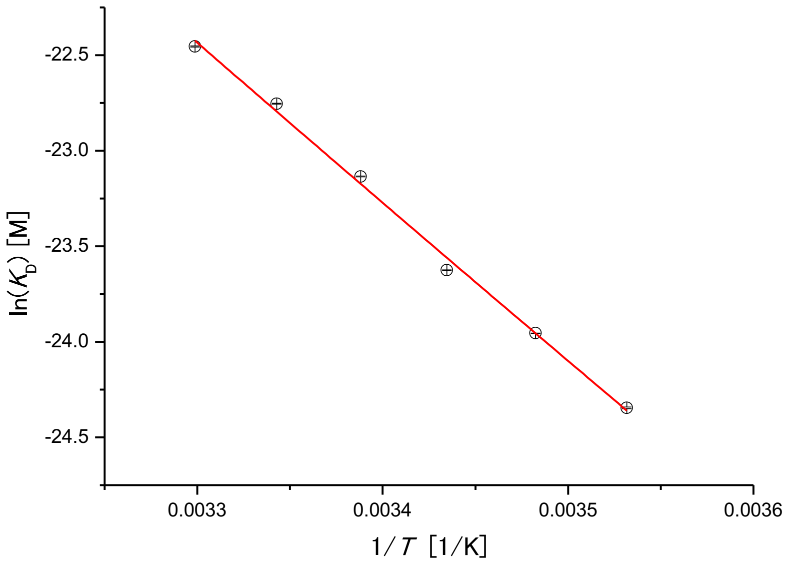

Supplementary Information Figure S2. Non-linear van't Hoff plot corresponding to the interaction of D3-L11 with HEL. Dissociation constants were determined at 10, 14, 18, 22, 26, and 30 °C. Bars indicate the standard error.

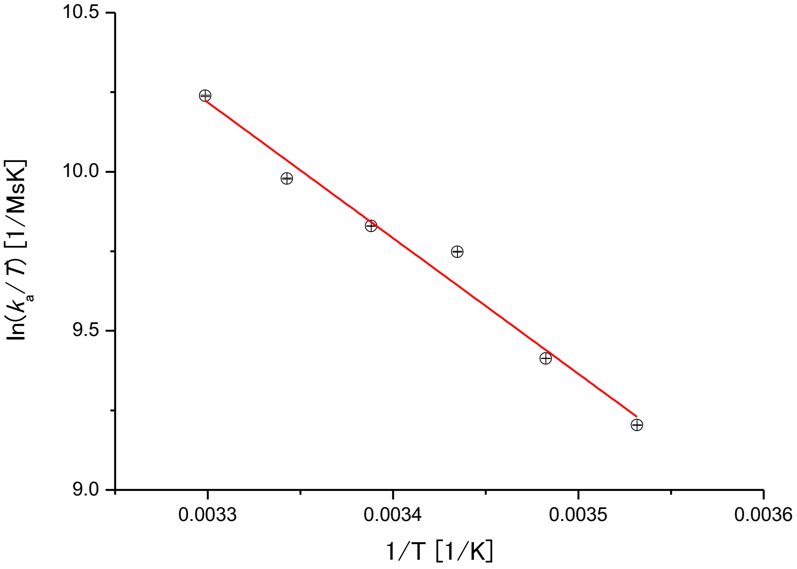

Supplementary Information Figure S3. Transition state analysis. The Eyring plot corresponding to the interaction of D3-L11 with HEL was obtained using the values of k_{on} determined at 10, 14, 18, 22, 26, and 30 °C in Fig. S1. Bars indicate standard error.

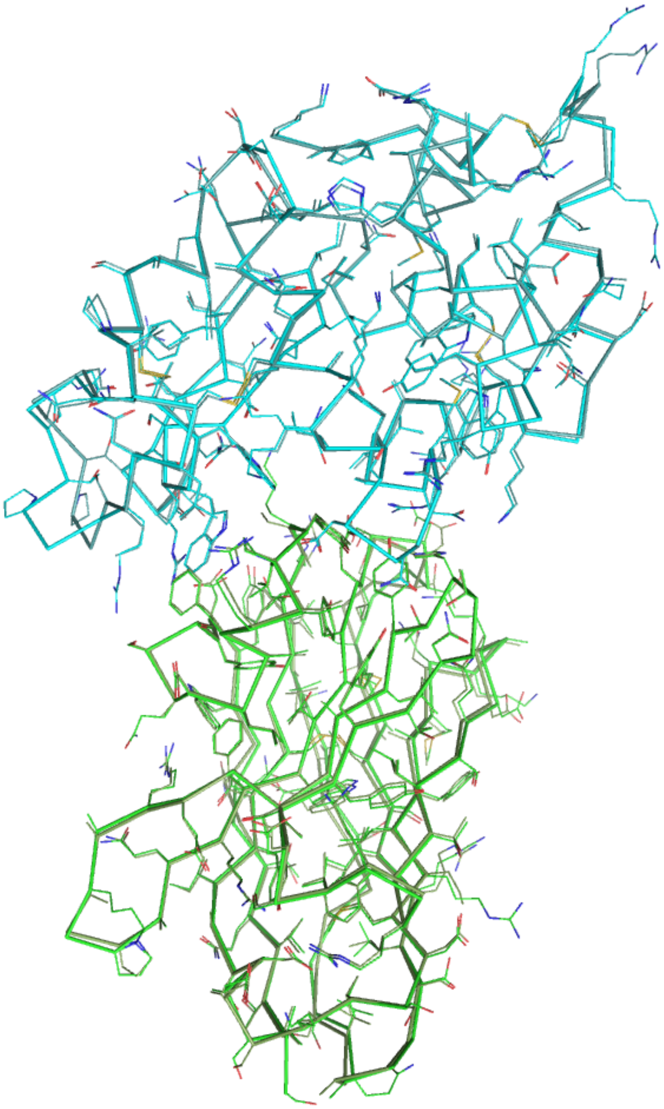

Supplementary Information Figure S4. Crystal structure of D3-L11 in complex with HEL. Antibody and HEL are depicted in green and cyan, respectively. The main chain was aligned to a previously reported crystal structure of the same complex crystallized in a different condition (PDB ID: 1ZVY). In the reported structure D3-L11 and HEL are shown in pale green and pale cyan, respectively

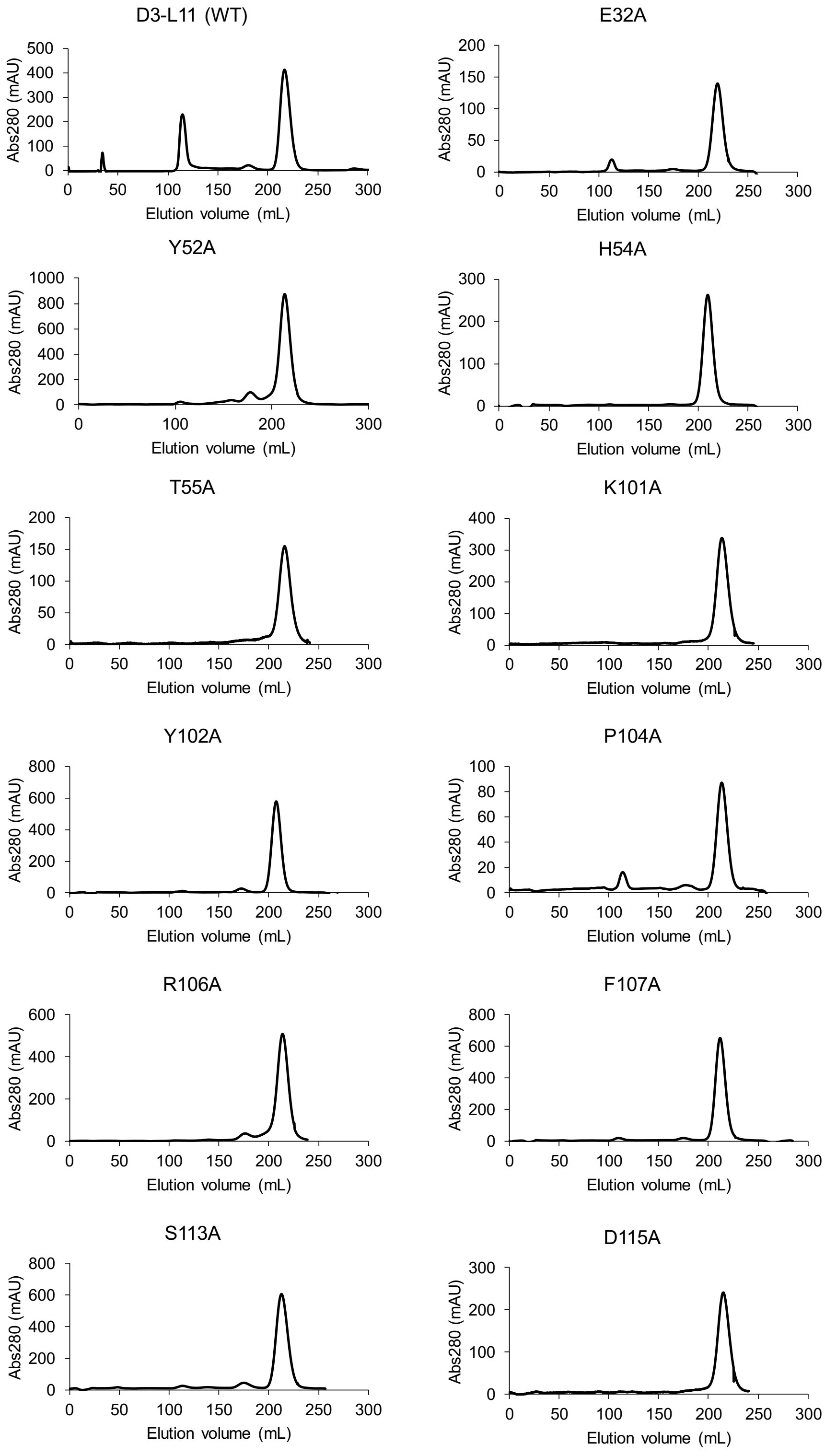

Supplementary Information Figure S5. Purification of D3-L11. Elution profile of WT antibody (and mutants) obtained by SEC. The peaks corresponding to monomeric VHH appeared at an elution volume of approximately 210 ml.

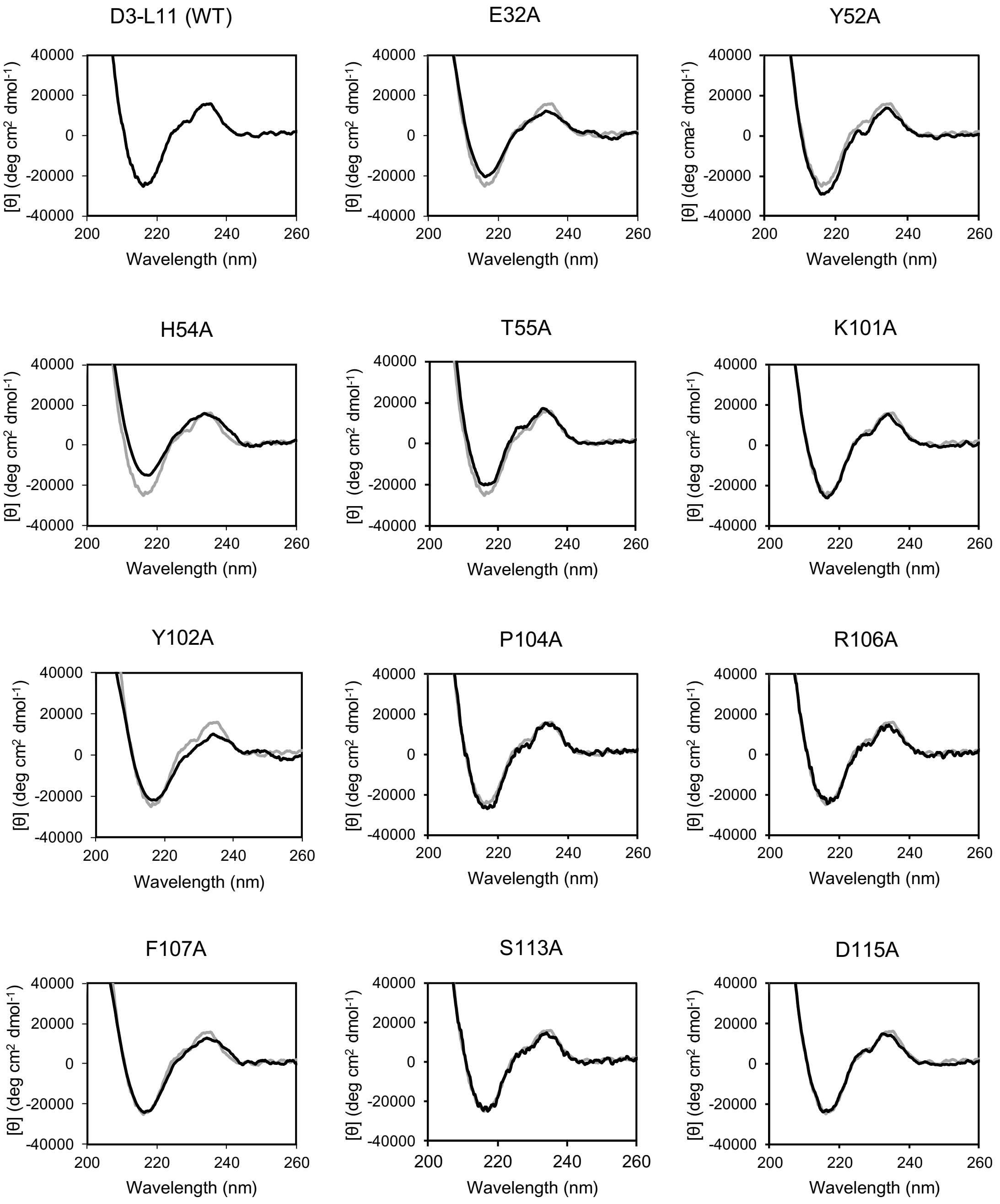

Supplementary Information Figure S6. CD spectra of WT D3-L11 and its mutants. Black and gray traces correspond to mutant and WT antibody, respectively, except for the top-left panel where WT is shown in black.

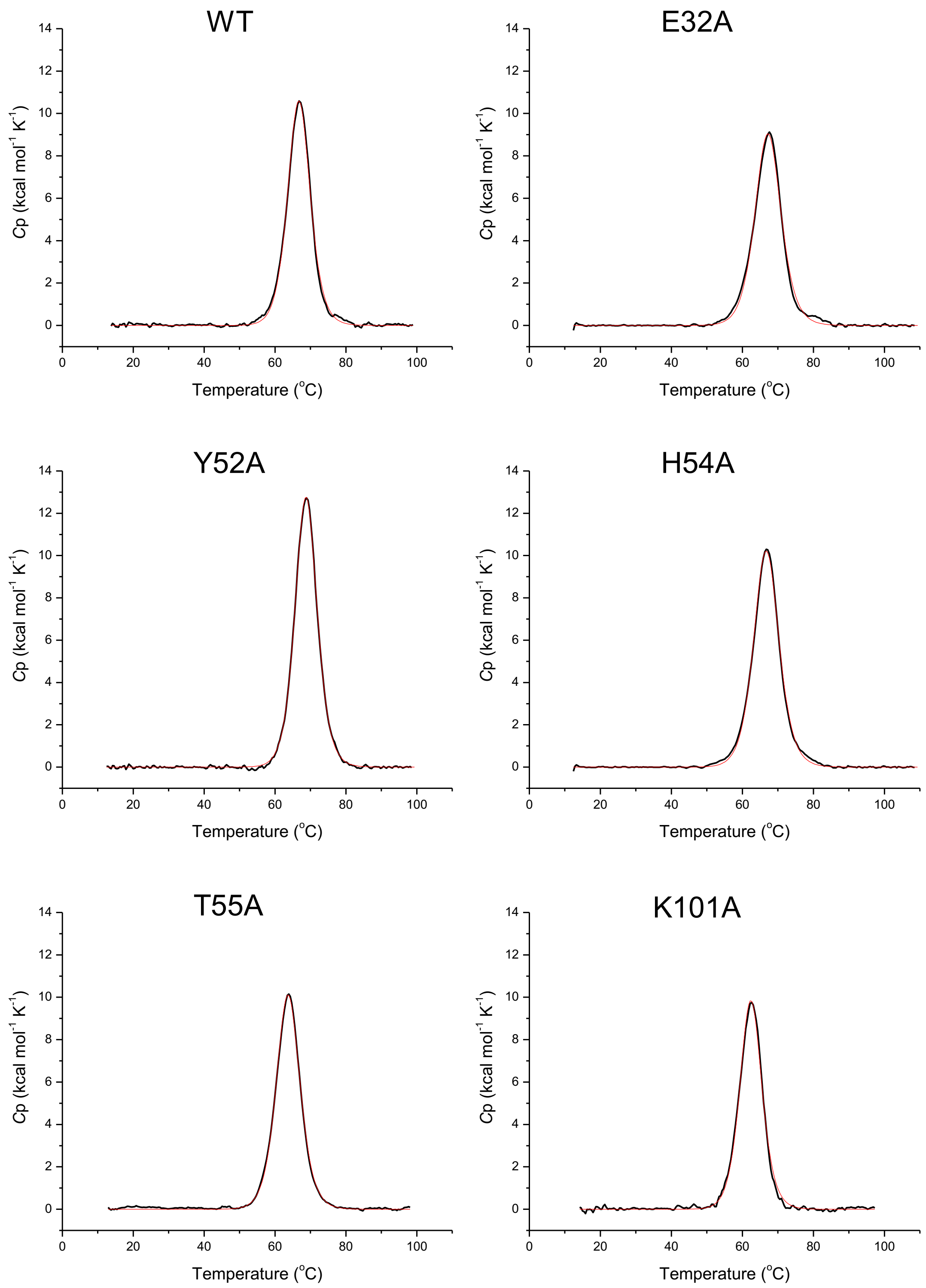

(Continues on the next page)

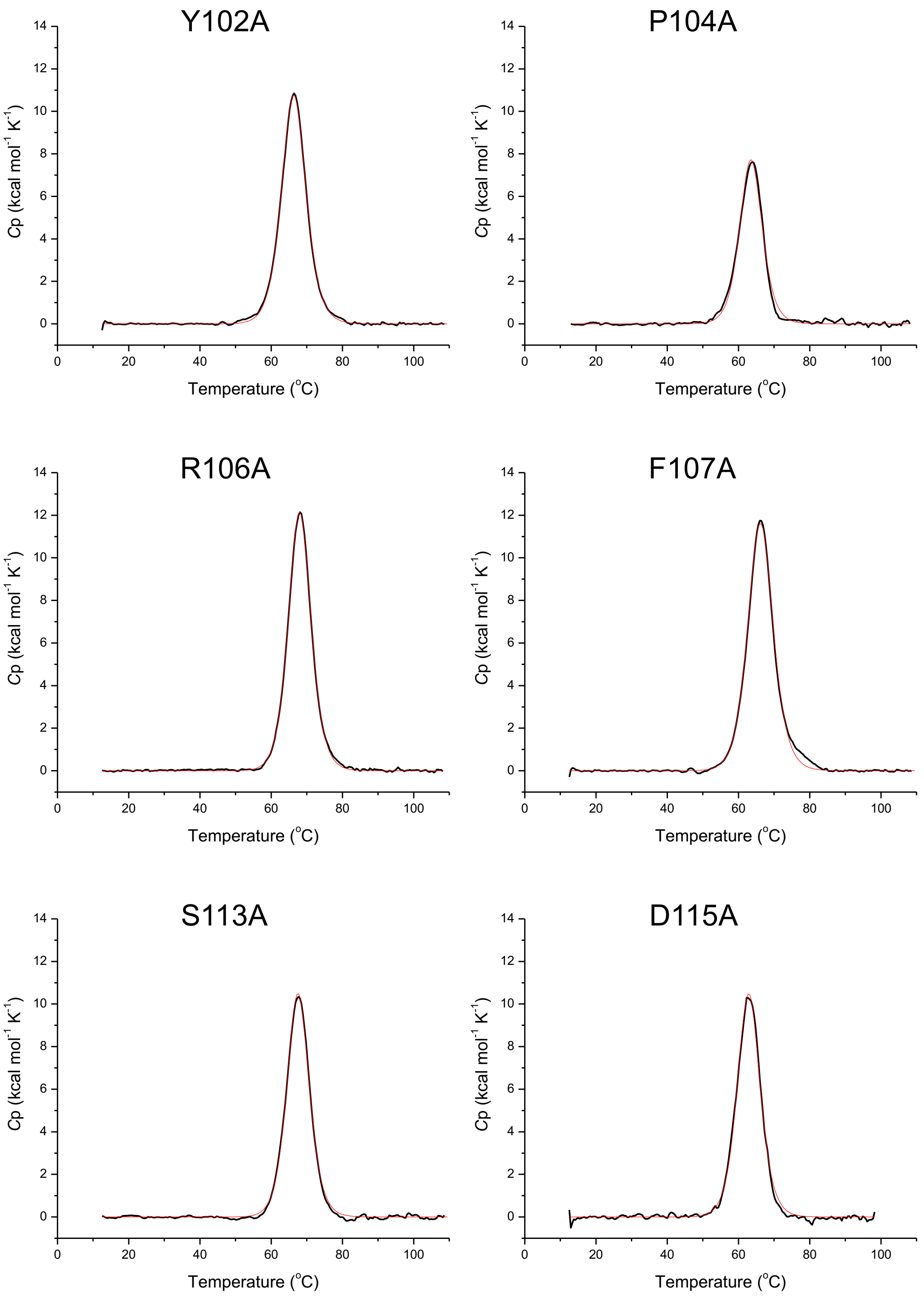

Supplementary Information Figure S7. DSC thermograms of WT D3-L11 and its mutants. The black and red traces correspond to the raw data and the best fitting to a non-two-state model, respectively.

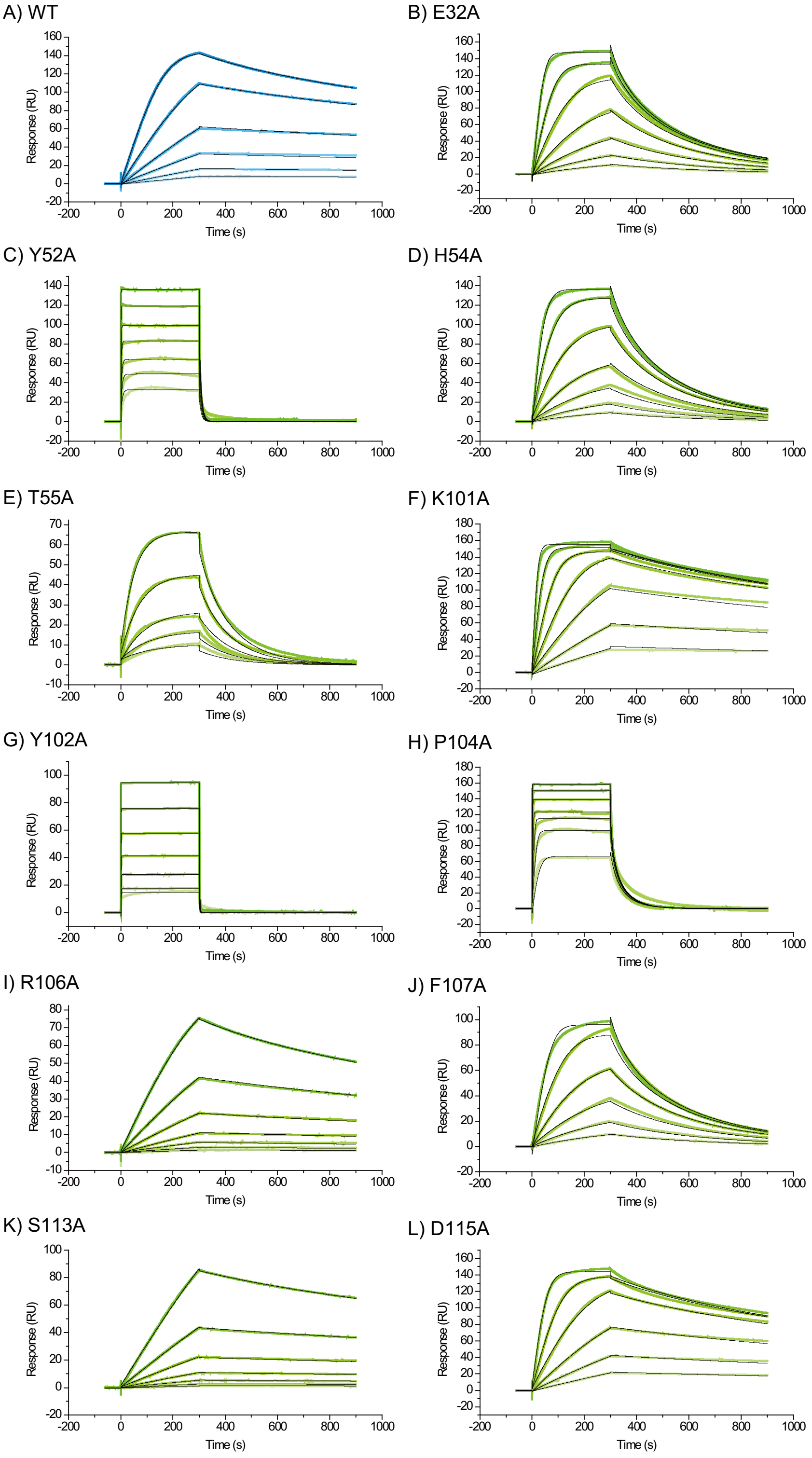

Supplementary Information Figure S8. SPR sensorgrams corresponding to the binding of D3-L11 to HEL at 25 ºC. Antibody was prepared in two-fold dilution series. Concentration range was: A) 2 nM – 0.125 nM; B, D, F, I, K) 16 nM – 0.25 nM ; C, H) 1024 nM – 16 nM; E) 4 nM – 0.25 nM; G) 2048 nM – 32 nM; J, L) 8 nM – 0.25 nM.

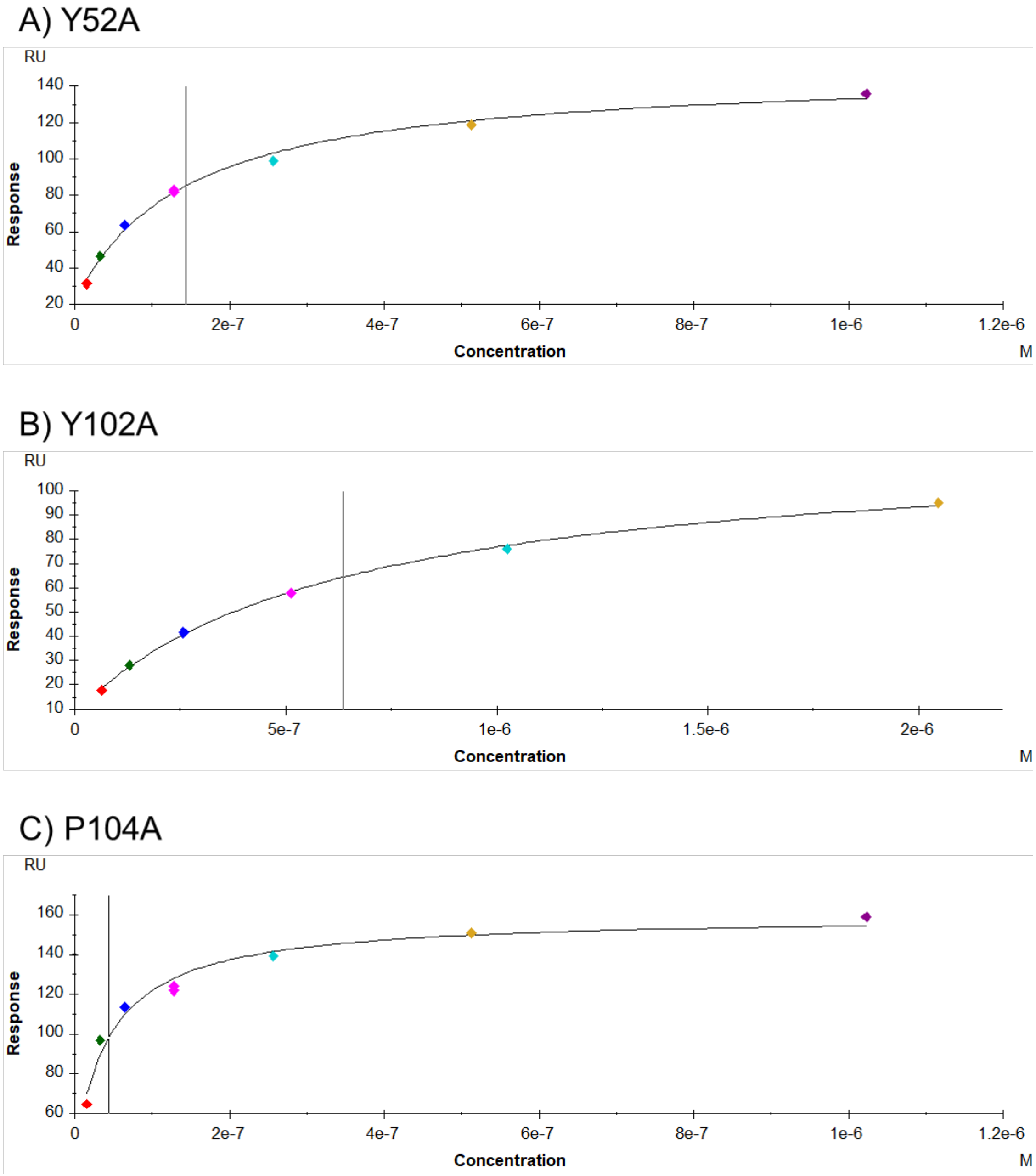

Supplementary Information Figure S9. Scatchard plot corresponding the maximal steady-state response at each concentration of antibody tested. The panels corresponded to three different mutants of D3-L11: A) Y52A, B) Y102A and C) P104A.

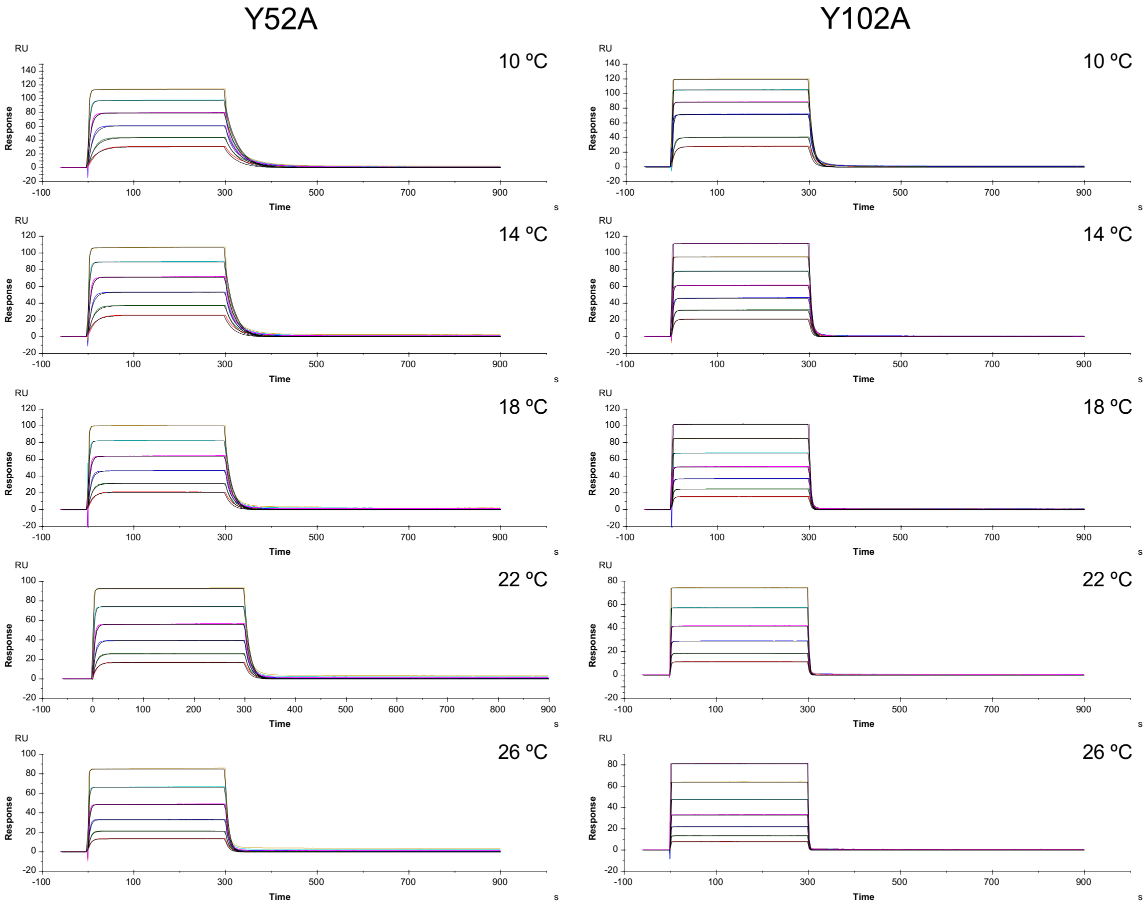

Temperature (ºC)	Y52A			Y102A		
	K_{on} ($\times 10^6$ $M^{-1}s^{-1}$)	K_{off} ($\times 10^{-3}$ s^{-1})	K_D (nM)	K_{on} ($\times 10^6$ $M^{-1}s^{-1}$)	K_{off} ($\times 10^{-3}$ s^{-1})	K_D (nM)
10	1.74 ± 0.01	37.0 ± 0.2	21.2 ± 0.2	1.15 ± 0.01	116 ± 0	100 ± 1
14	1.94 ± 0.01	51.5 ± 0.2	26.5 ± 0.2	1.38 ± 0.01	201 ± 0	146 ± 1
18	2.21 ± 0.01	73.1 ± 0.2	33.0 ± 0.2	1.62 ± 0.01	309 ± 1	191 ± 1
22	2.50 ± 0.01	102 ± 0	40.8 ± 0.2	2.06 ± 0.02	479 ± 4	233 ± 3
26	2.72 ± 0.02	144 ± 1	52.9 ± 0.4	1.99 ± 0.01	830 ± 2	418 ± 2

Supplementary Information Figure S10. Sensorgrams corresponding to the interaction of HEL with mutants Y52A or Y102A of D3-L11 at various temperatures. The values of the association and dissociation rates, and those of the dissociation constants are given in the lower panel with standard error of the curve fitting.

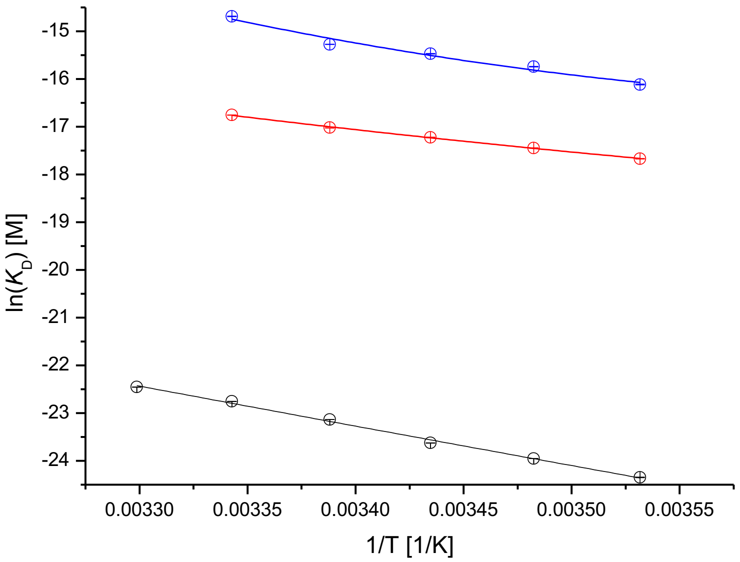

Supplementary Information Figure S11. Non-linear van't Hoff plot corresponding to the interaction of D3-L11 with HEL. The dissociation constants were determined at 10, 14, 18, 22, and 26 °C using the sensorgrams in Fig. S1 and S10. Black, red and blue colors correspond to WT, Y52A and Y102A, respectively. Bars indicate the standard error.

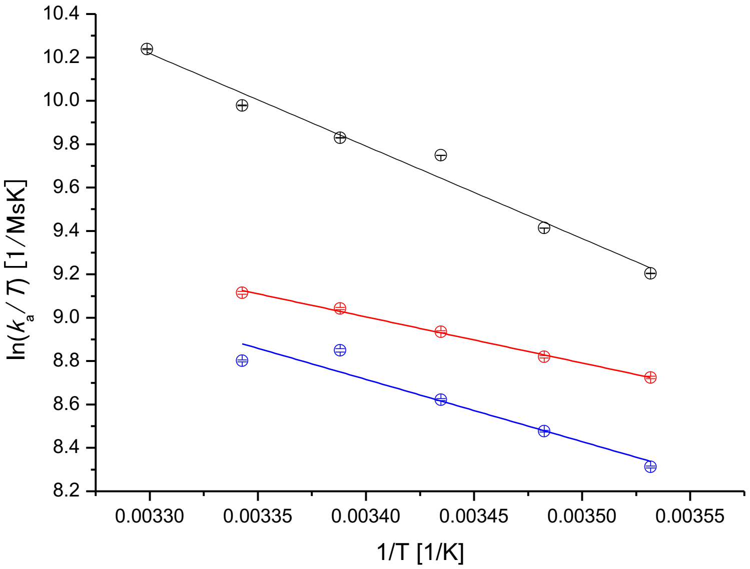

Supplementary Information Figure S12. Transition state analysis. The Eyring plot corresponding to the interaction of D3-L11 and mutants with HEL was obtained using the values of k_{on} determined at 10, 14, 18, 22 and 26 °C in Figs S1 and S10. Black, red and blue data correspond to WT, Y52A and Y102A, respectively. Bars indicate the standard error.

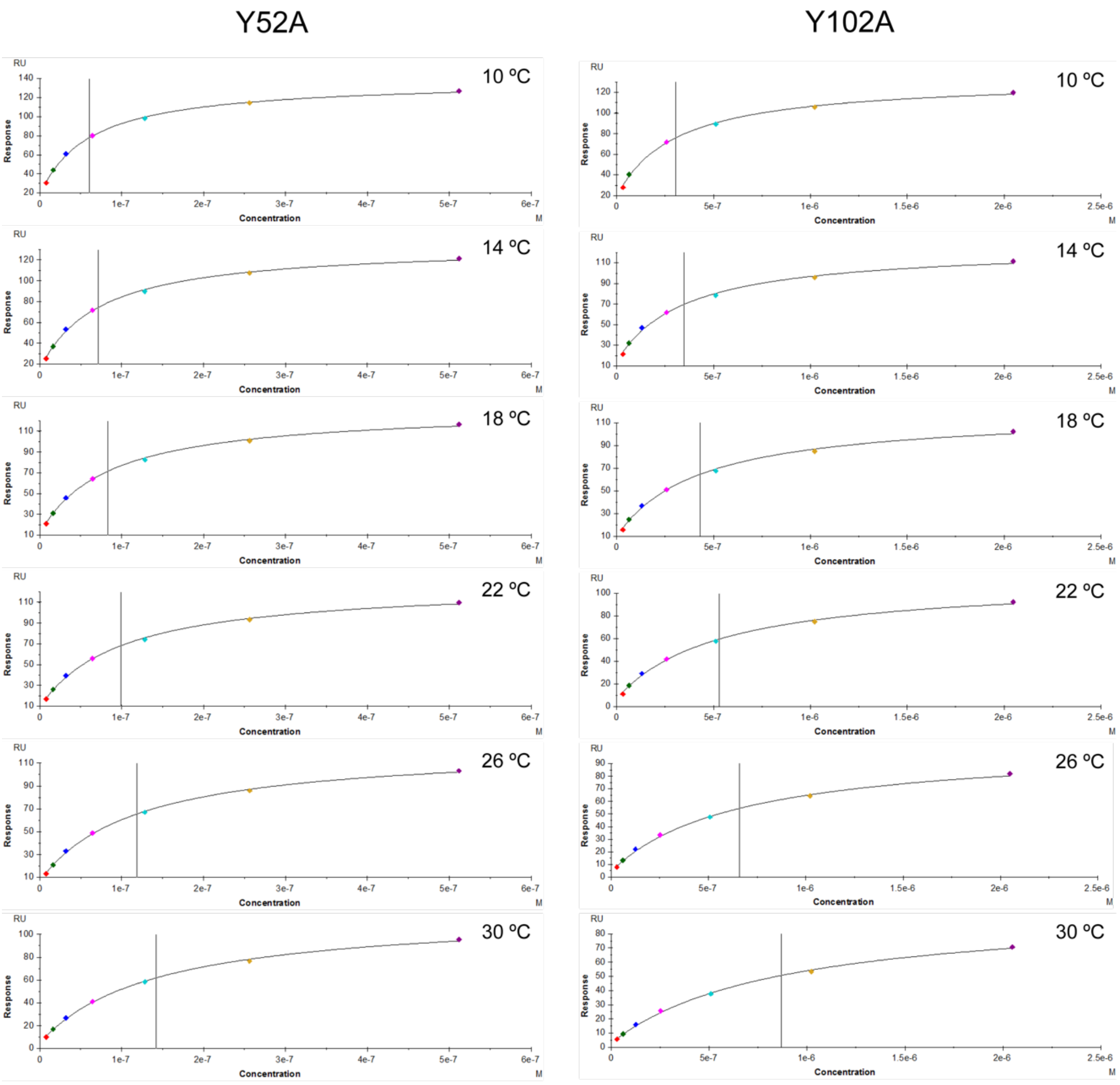

ºC	K_D (nM) Y52A	Y102A
10	60.5 ± 4.3	303 ± 32
14	71.4 ± 5.3	348 ± 42
18	83.4 ± 6.5	429 ± 48
22	99.2 ± 7.4	529 ± 55
26	119 ± 9	658 ± 64
30	142 ± 10	867 ± 70

Supplementary Information Figure S13. Scatchard plot and corresponding dissociation constants for the interaction of Y52A and Y102A with HEL at six different temperatures.

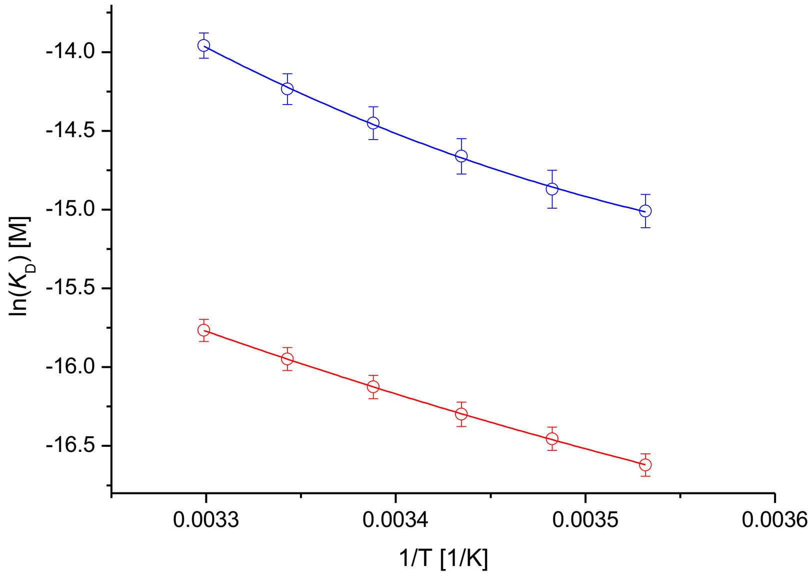

Supplementary Information Figure S14. Non-linear van't Hoff plot corresponding to the interaction of mutants of Y52A and Y102A with HEL. The plot is based on the dissociation constants obtained from the Scatchard plots at 10, 14, 18, 22, 26 and 30 °C (see Fig. S13). Black and red correspond to Y52A and Y102A, respectively. Bars indicate the standard error.

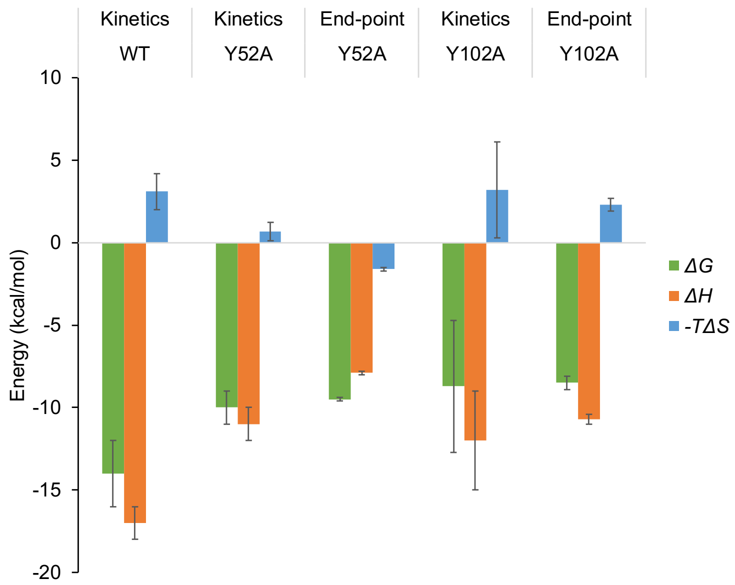

Supplementary Information Figure S15. Thermodynamic parameters determined from the van't Hoff plots in Figs S11 (global analysis of multi-cycle sensorgrams) and S14 (Scatchard plot).

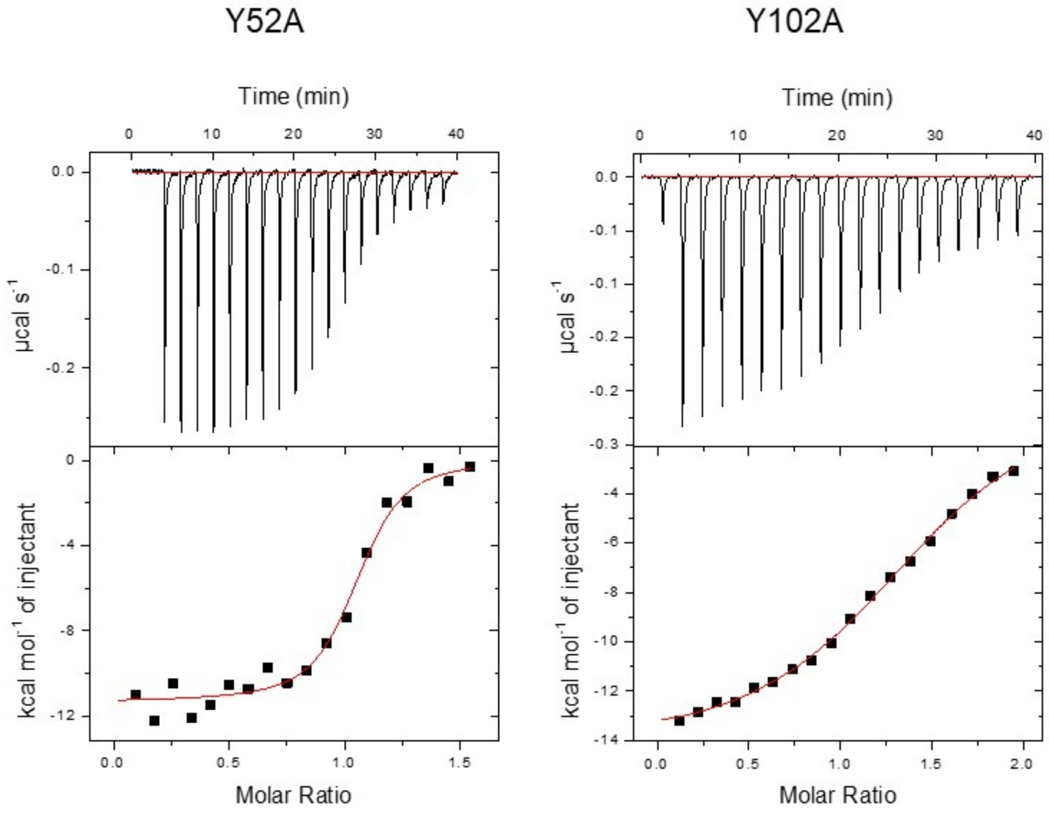

Supplementary Information Figure S16. Binding between mutant Y52A or mutant Y102A and HEL. The top panels correspond to the titration traces and the bottom panels to the binding isotherm integrated from the titration curves.

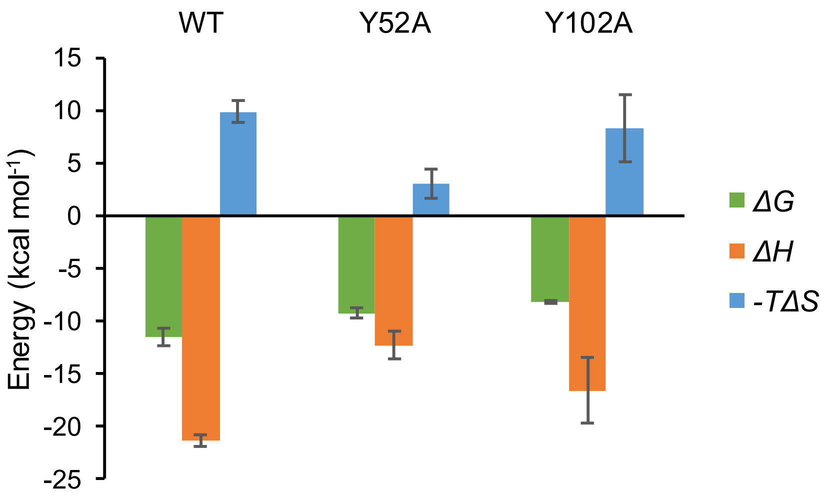

Supplementary Information Figure S17. Thermodynamic parameters of the binding of HEL to D3-L11 WT and mutants determined by ITC. Error bars indicate the standard error from two independent experiments.
